# A Stoichiometric Solvent-Free Protocol for Acetylation Reactions

**DOI:** 10.3389/fchem.2022.842190

**Published:** 2022-03-09

**Authors:** Francesca Valentini, Pierluca Galloni, Diana Brancadoro, Valeria Conte, Federica Sabuzi

**Affiliations:** ^1^ Department of Chemical Science and Technologies, University of Rome Tor Vergata, Rome, Italy; ^2^ BT-InnoVaChem Srl, Rome, Italy

**Keywords:** acetylation, acetic anhydride, isopropenyl acetate, sustainability, thymol, phenols, alcohols, thiols

## Abstract

Considering the remarkable relevance of acetylated derivatives of phenols, alcohols, and aryl and alkyl thiols in different areas of biology, as well as in synthetic organic chemistry, a sustainable solvent-free approach to perform acetylation reactions is proposed here. Acetylation reactions are classically performed using excess of acetic anhydride (Ac_2_O) in solvent-free conditions or by eventually working with stoichiometric amounts of Ac_2_O in organic solvents; both methods require the addition of basic or acid catalysts to promote the esterification. Therefore, they usually lead to the generation of high amounts of wastes, which sensibly raise the E-factor of the process. With the aim to develop a more sustainable system, a solvent-free, stoichiometric acetylation protocol is, thus, proposed. The naturally occurring phenol, thymol, can be converted to the corresponding—biologically active—ester with good yields, in the presence of 1% of VOSO_4_. Interestingly, the process can be efficiently adopted to synthesize other thymyl esters, as well as to perform acetylation of alcohols and aryl and alkyl thiols. Remarkably, a further improvement has been achieved replacing Ac_2_O with its greener alternative, isopropenyl acetate (IPA).

## Introduction

In the framework of organic transformations, acetylation is a common and versatile reaction, extensively used both on laboratory and industrial scale. In fact, acetate is usually exploited as an effective protective group for phenols, alcohols, thiols, and amines in several multistep syntheses, including drug preparation (Carey et al., 2006). Moreover, acetylation of bioactive molecules, such as natural phenols, confers enhanced lipophilicity, eventually leading to an improved bioactivity (Su et al., 2020; Floris et al., 2021). The key role of such a reaction is particularly highlighted in the case of Aspirin^®^, where acetylation is fundamental to allow safe drug administration (Vane and Botting, 2003). Therefore, it is particularly important to find sustainable methodologies to perform acetylation reactions in good yields. Acetic anhydride (Ac_2_O) is amongst the most used acetylating reagent (Larionov and Zipse, 2011), but basic or acid catalysts are needed to activate it. Yet, 4-(dimethylamino)pyridine (DMAP) (Steglich and Hofle, 1969; Mandai et al., 2018), triethylamine, and tributylphosphine (Vedejs et al., 1993) are generally used as basic catalysts for Ac_2_O activation in chlorinated solvents. Analogously, several Lewis acid complexes with hard anions (Chandra et al., 2002; Reddy et al., 2006) that significantly enhance metal acidity, such as triflates (Ishihara et al., 1995; Barrett and Braddock, 1997; Chauhan et al., 1999; Saravanan and Singh, 1999; Chen et al., 2001; Orita et al., 2001; Das and Chakraborty, 2011; Kumar et al., 2014), perchlorates (Bartoli et al., 2003), and chlorides (De, 2004), have been adopted in acetylation reactions with Ac_2_O, showing high catalytic activity. Interestingly, acetylation of tertiary alcohols, which generally show slow rates and unsatisfactory yields, has been successfully obtained by merging together the catalytic activity of Sc(OTf)_3_ and DMAP (Zhao et al., 1998). However, several disadvantages are associated with these catalysts, such as the moisture sensitivity as well as their high prices. Furthermore, chlorinated solvents, high reaction temperatures, and inert atmosphere are usually required.

In the last years, the chemistry community has been committed to find new sustainable approaches to perform organic transformations, preferring environment-friendly methodologies. Such task results particularly relevant for industrial processes (Sarkar et al., 2016). In this respect, acetylation of phenols, alcohols, amines, and thiols has been extensively investigated, with the goal of improving sustainability through homogenous (Lugemwa et al., 2013; Singha and Ray, 2016; Temperini et al., 2017; Kuciński and Hreczycho, 2018; Zhu et al., 2018; Jain et al., 2019; Chutia and Chetia, 2020; Pantawane et al., 2021) and heterogenous catalysis (Rajabi and Luque, 2014; Bajracharya and Shrestha, 2018; Behera and Patra, 2021; Hlatshwayo et al., 2021). Indeed, earth-abundant transition metals (Zhu et al., 2018; Jain et al., 2019), solvent-free conditions (Rajabi and Luque, 2014; Behera and Patra, 2021), and safe acylating reagents (Singha and Ray, 2016; Temperini et al., 2017; Pantawane et al., 2021) have been employed. Among transition metal complexes, vanadium covers a chief role as an environment-friendly catalyst for several organic transformations, including oxidation and halogenation reactions (Conte and Floris, 2010; Galloni et al., 2013; Floris et al., 2017; Coletti et al., 2018; Sabuzi et al., 2019; Sabuzi et al., 2021; Valentini et al., 2021). Moreover, various inorganic and organic vanadium (IV) complexes have been studied as catalysts in acetylation of alcohols, thiols, and amines, with high excess of acetic anhydride, eventually adding organic solvents (Chen et al., 2001; Oskooie et al., 2008; Taghavi et al., 2011).

In this study, vanadium (IV)-based catalysts have been employed in the sustainable esterification of thymol (2-isopropyl-5-methylphenol, 1), a natural phenolic compound, particularly known at the industrial level for its peculiar biological properties. Notably, functionalization allows to access different thymol derivatives characterized by even more improved bioactivity (Galloni et al., 2018; Piombino et al., 2020; Floris et al., 2021). To this purpose, over the last years, we have been involved in the synthesis of differently functionalized thymol-based products for applications in the biological and cosmetical fields (Sabuzi et al., 2015; Galloni et al., 2018; Piombino et al., 2020). In addition, many studies demonstrated that thymyl acetate resulted more effective than thymol against different pathogenic fungi and several bacterial strains (Floris et al., 2021); other thymyl esters have been recently highlighted for their promising biological and pharmacological activities (Chauhan et al., 2017; Tharamak et al., 2020; Floris et al., 2021). Therefore, considering the growing interest in such valuable compounds, a sustainable method for thymol esterification, even extended for the esterification of other phenols, aliphatic alcohols, and thiols, is presented here.

## Methods

All commercial reagents and solvents were purchased from Sigma-Aldrich/Merck Life Science, with the highest degree of purity, and they were used without any further purification. GC-MS analyses have been performed with a Shimadzu GCMS QP2010 Ultra system. ^1^H-NMR experiments have been performed with Bruker Avance 700 MHz.

### General Procedure for the Synthesis of Thymyl Acetate and Esters 3a–d

In a round-bottom flask, VOSO_4_·5H_2_O was suspended in the proper volume of the anhydride. After 10 min, 6.6 mmol (1 g) of thymol was added. The reaction was kept under stirring for 2 or 24 h at room temperature. The reaction was quenched with the addition of 50 ml of distilled H_2_O, and it was kept under stirring for about 15 min. 10 ml of NaHCO_3_ (s s.) were then added to the aqueous phase to neutralize the carboxylic acid formed as by-product. The aqueous phase was extracted with 100 ml of ethyl acetate. The organic phase was washed with 100 ml of water until neutrality was reached, dried over anhydrous Na_2_SO_4_, and filtered, and the solvent was removed under reduced pressure. The oil was purified by using a chromatography column (SiO_2_, DCM: petroleum ether 2:3 *v:v*).

### Synthesis of Thymyl Acetate on a 10 g Scale

168 mg of VOSO_4_·5H_2_O (0.66 mmol) was suspended in 6.4 ml of Ac_2_O (0.068 mol). After 10 min, 10 g of thymol (0.067 mol) was added, and the reaction was kept under stirring for 24 h at room temperature. 150 ml of H_2_O was added, and the aqueous phase was extracted with 2 × 100 mL of ethyl acetate. The organic phase was washed with 2 × 50 mL of 1 M NaOH solution to remove unreacted thymol and then with water. The organic phase was dried over anhydrous Na_2_SO_4_ and filtered, and the solvent was evaporated. The product was obtained as a colorless oil. Purity was checked by TLC and ^1^H NMR analyses. Yield = 87% (11.2 g, 0.058 mol).

### Synthesis of Thymyl Acetate on a 50 g Scale

50 g of thymol (0.33 mol) was added to a solution of 32 ml of acetic anhydride (0.34 mol) containing 835 mg of VOSO_4_·5H_2_O (3.3 mmol). The reaction was kept under stirring for 24 h at room temperature. The reaction was quenched with the addition of 200 ml of H_2_O. Being non-soluble in water, the synthesized product has been separated from the aqueous phase through a separatory funnel. The product was diluted with 50 ml of ethyl acetate extracted with 2 × 50 mL of 1 M NaOH solution to remove unreacted thymol and then with water. Then, it was dried over anhydrous Na_2_SO_4_ and filtered, and the solvent was removed under reduced pressure. The product was obtained as a colorless oil. Purity was checked by TLC and ^1^H NMR analyses. Yield = 97% (62.3 g, 0.32 mol).

### Synthesis of Esters and Thioesters 5a–h

In a 5 ml round-bottom flask, 1 g of 4a–h was added to an equimolar amount of acetic anhydride containing 1% of VOSO_4_·5H_2_O. The reaction was kept under stirring for 2 or 24 h at room temperature. The reaction was then quenched with 50 ml of H_2_O and kept under stirring for about 15 min. 10 ml of NaHCO_3_ (s s.) was then added to the aqueous phase, and it was extracted with 100 ml of ethyl acetate. The organic phase was then washed with 100 ml of distilled water until neutrality was reached. The organic phase was dried over anhydrous Na_2_SO_4_, filtered, and evaporated. The obtained product was purified by using a chromatography column.

Products have been characterized with GC-MS, ^1^H NMR, and ^13^C-NMR analyses. Data are included in Supplementary Material S1.

### General Procedure for *O*-Acetylation With Isopropenyl Acetate

In a 5 ml round-bottom flask, 1% of VOSO_4_·5H_2_O was dissolved in 1 eq. of isopropenyl acetate. After 10 min, 1 g of substrate was added. The reaction was kept under magnetic stirring at 60°C for 24 h. An aliquot of 4 μL was taken and diluted in 5 ml with a solution 10 mM decane in ethyl acetate and analyzed with GC-MS. Results are reported in Supplementary Table S7 and Figure 3.

## Results and Discussion

Thymol acetylation was initially explored using acetic anhydride as an acetylating reagent. Ac_2_O activation was promoted by V(IV)-based catalysts, namely, vanadyl sulfate (VOSO_4_) and vanadyl acetylacetonate (VO(acac)_2_). To minimize waste production, thus increasing process sustainability, reactions have been carried out in solvent-free conditions. Consequently, Ac_2_O has been exploited as a reagent and solvent. Results are reported in Table 1.

**TABLE 1:** Thymol acetylation. Reaction conditions: thymol = 6.7 mmol (1 g), T = r. t., and t = 24 h.

Entry	V-Cat	V-Cat (%)	Ac_2_O (eq)	Isolated Yield (%)	E-factor^d^
1	VOSO_4_	5%	8	85	8.7
2	VO(acac)_2_	5%	8	85	8.7
3	VOSO_4_	1%	8	80	9.2
4	VO(acac)_2_	1%	8	74	10.0
5	VOSO_4_	0.5%	8	71	10.5
6	VOSO_4_	1%	3.2	85	5.6
7	VOSO_4_	1%	1.2	83	4.5
8	VOSO_4_	1%	1.0	83	4.4
9^a^	VOSO_4_	1%	1.0	87	1.3
10^b^	VOSO_4_	1%	1.0	97	0.6
11	—	—	1.0	3^c^	146.9

a Reaction performed on 10 g of thymol.

b Reaction performed on 50 g of thymol.

c GC-MS yield.

d Solvent contribution not included (Sabuzi et al., 2015).

Results show that reactions performed at room temperature, with 5% of VOSO_4_ or VO(acac)_2_ and 8 equivalents of acetic anhydride, led to 85% of isolated product, and no difference between the catalysts has been observed (entries 1–2). Catalyst loading was then decreased to 1% (entries 3–4), and VOSO_4_ showed higher catalytic performance than VO(acac)_2_. In particular, thymyl acetate was obtained with 80% yield in 24 h at room temperature, and a further decrease of the catalyst to 0.5% led to only a slight decrease of the yield (entry 5). Thus, 1% of vanadyl sulfate has been chosen for further experiments. Interestingly, with respect to the other catalysts commonly used for acetylation reactions such as amines (Steglich and Hofle, 1969; Mandai et al., 2018), phosphines (Vedejs et al., 1993), and Lewis acid complexes with hard anions (Chandra et al., 2002; Reddy et al., 2006), environmental and human health risks associated with the use of VOSO_4_ are reduced, being non-toxic for humans, non-flammable, or non-corrosive. Therefore, VOSO_4_ is considered a safe and biocompatible catalyst; recently, the pharmacological benefits associated with its assumption have been highlighted (Ścibior et al., 2020).

In order to further improve the sustainability of such process, the amount of Ac_2_O was gradually decreased, and its effect on product yield has been investigated (entries 6–8): reduction of Ac_2_O up to 1 equivalent (620 μL per gram of thymol) does not affect product yield, which still results higher than 80% (entry 8). The remarkable progress reported here is the VOSO_4_ reduction to 1% and the stoichiometric amount of Ac_2_O. In fact, in previously optimized processes, 50% excess of the reactant and higher catalyst loadings (5% mol) were required (Oskooie et al., 2008). Such a result greatly improves the E-factor (Sheldon, 2008) of the process, since a 1:1 stoichiometric ratio between the reactants is preferred to avoid large amount of wastes (Sarkar et al., 2016). Interestingly, reactions performed on a larger scale (i.e*.*, on 10 and 50 g of substrate) led to even more improved yields and strongly reduced E-factor values because of the simplified workup (entries 9–10). In particular, 97% of thymyl acetate was isolated performing the reaction on a 50 g scale, likely anticipating promising results for further scale-ups. To note, all the reactions led to the selective formation of thymyl acetate, and blank experiments showed only 3% of the product (entry 11).

**FIGURE 1:**
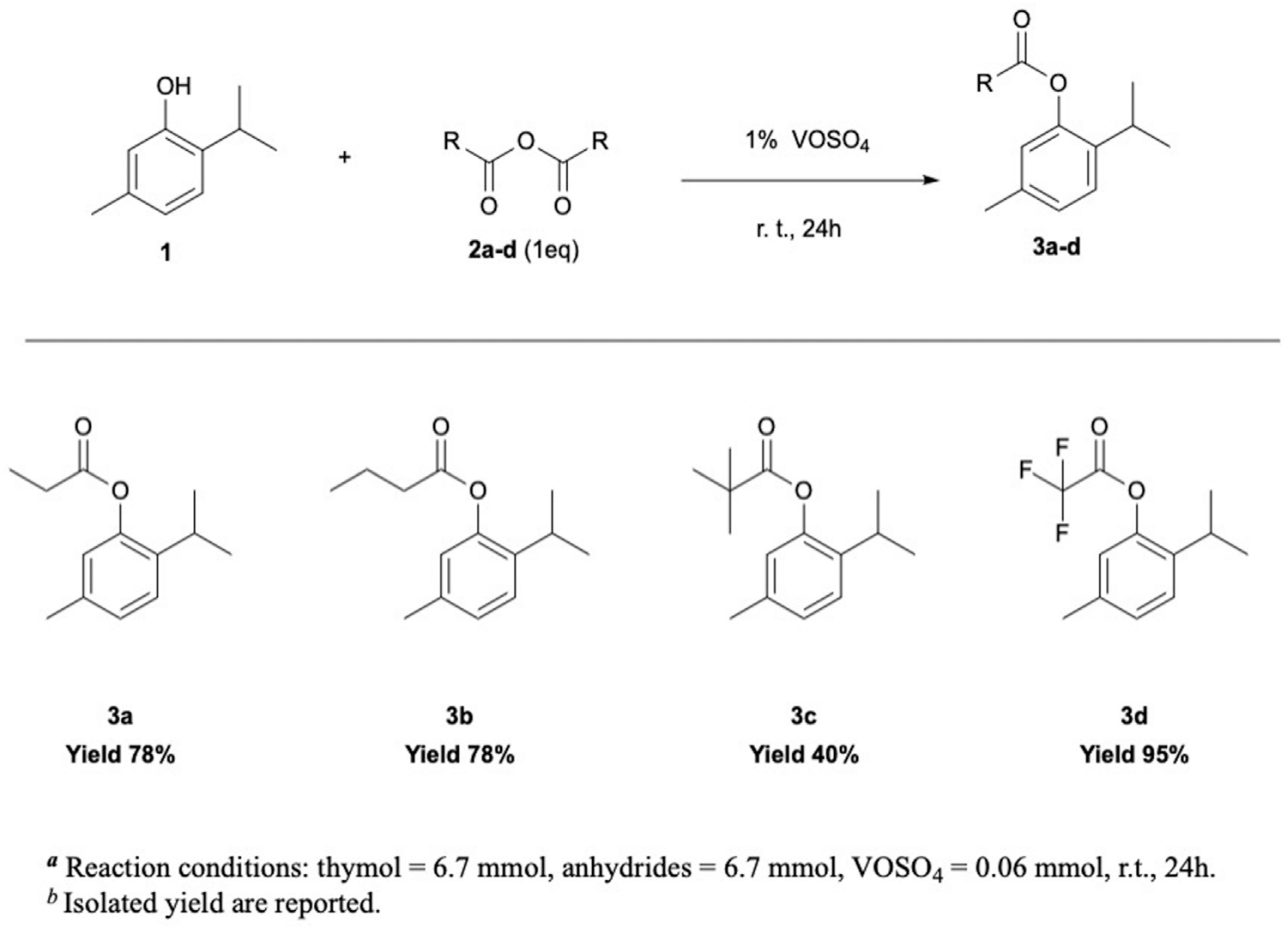
Thymol acetylation reaction using different anhydrides as the acetylating agent.

Considering the biological relevance of thymyl esters, the optimized reaction conditions have been exploited to perform thymol esterification with different anhydrides (Figure 1). Remarkably, thymyl esters 3a–3d were obtained in moderate to very good yields at room temperature, working with a stoichiometric amount of reactants and 1% of VOSO_4_ (Supplementary Table S1). Notably, by increasing the acylating reagent side chain length, a slight decrease in product yield was observed (Supplementary Table S1, entries 1–4), while with trimethyl acetic anhydride (TMA) (Supplementary Table S1, entries 5–6), only 40% of the product was obtained after 24 h. As a matter of fact, both steric hindrance and inductive effect of the three alkyl groups likely deactivate TMA toward a nucleophilic attack (Chen et al., 2001). Conversely, trifluoroacetic anhydride resulted more reactive than the others; indeed, a significant increase in the yield was observed, reaching 90% of isolated product in 2 h and 95% in 24 h (Supplementary Table S1, entries 7–8).

**FIGURE 2:**
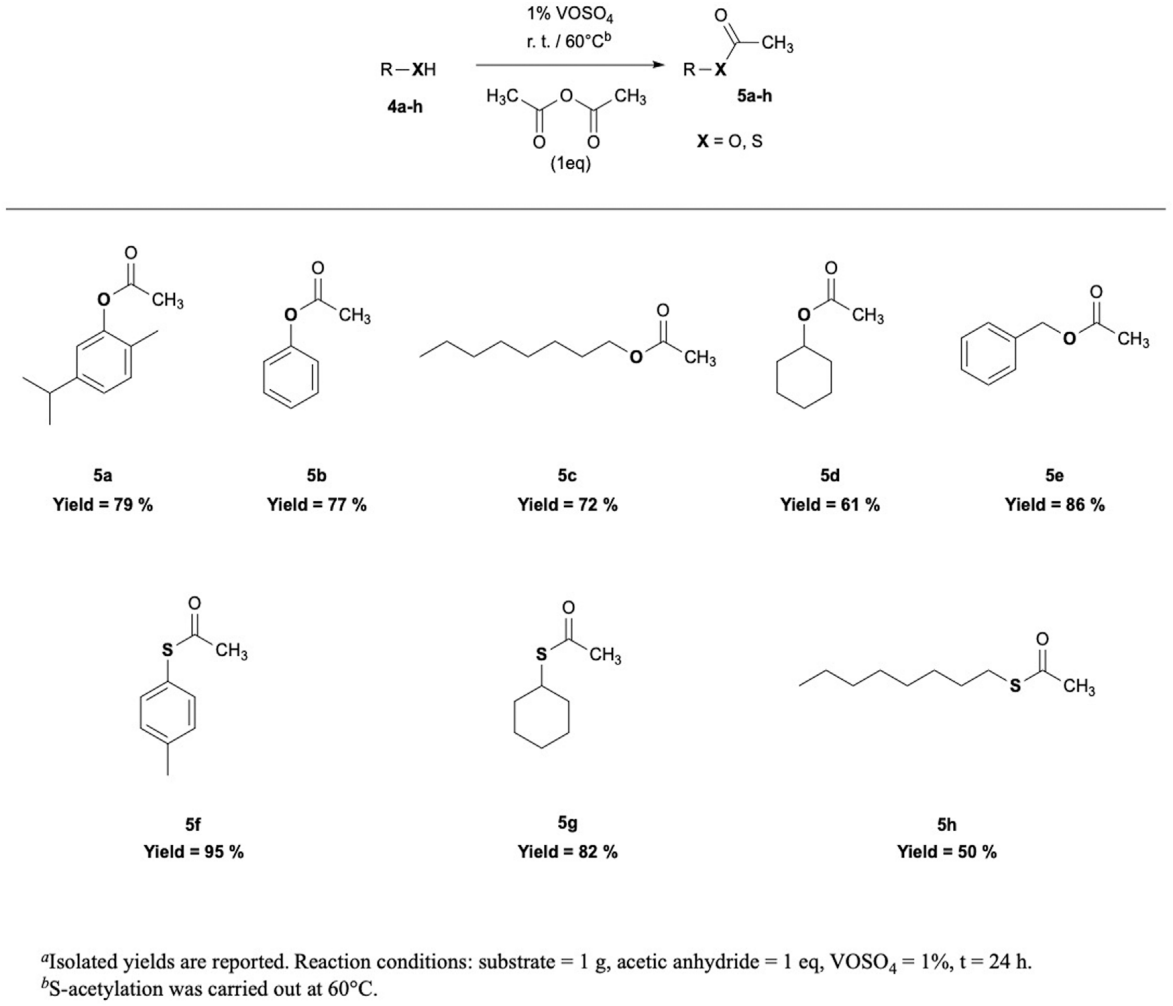
*O,S*-acetylation reactions: substrate scope investigation.

Given the very good results achieved in thymol esterification, substrate scope has been investigated using acetic anhydride as a model reagent. *O-* or *S*-acetylation of phenols, alcohols, and aryl and alkyl thiols has been performed, using a stoichiometric amount of acetic anhydride and 1% of vanadyl sulfate (Figure 2, Supplementary Tables S3, S4).

Carvacryl and phenyl acetates (5a–b) were obtained with good yields, comparable to that of thymyl acetate in the same conditions, and 86% yield was achieved with benzyl acetate (5e) in 24 h at room temperature. Also, 1-octanol and cyclohexanol were converted to their corresponding esters (5c–d) with a good but slightly lower yield. On the contrary, *S*-acetylation of alkyl and aryl thiols required higher reaction temperature (Supplementary Table S4). Still, 4-methylphenyl thioacetate (5f) has been obtained with 95% yield at 60°C, while alkyl thiols, such as cyclohexanethiol and 1-octanethiol, were converted to their corresponding thioacetate with 68 and 50% of isolated yield, respectively, likely indicating that both substrate nucleophilicity and steric hindrance affect reaction outcome.

**FIGURE 3:**
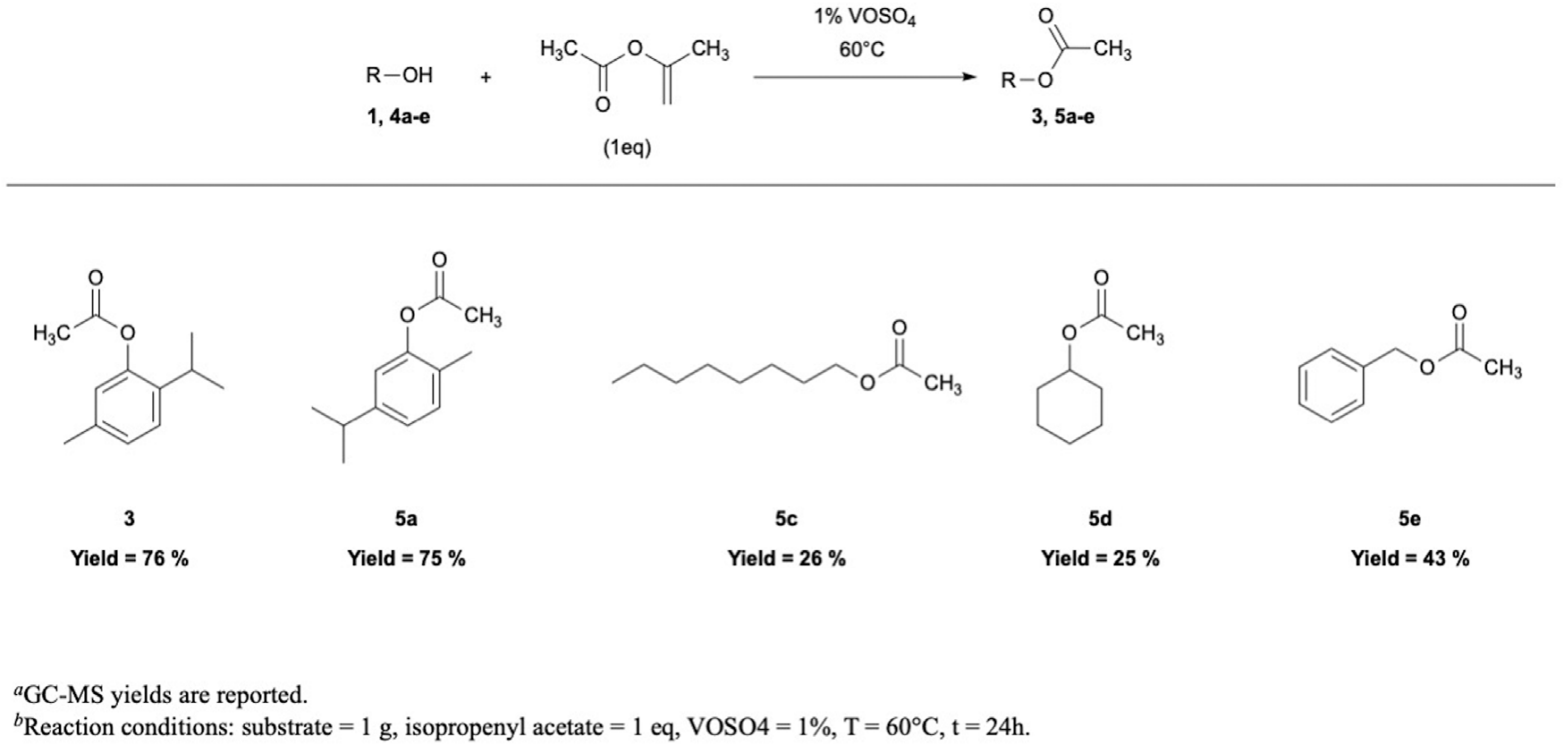
*O*-acetylation with isopropenyl acetate.

In the context of acetylation reactions, isopropenyl acetate (IPA) is recently emerging as a new green acetylating reagent for alcohols (Barry, et al., 1988; Temperini et al., 2017; Zhu et al., 2018; Rigo et al., 2020), thiols (Kuciński and Hreczycho, 2018), and amines (Pelagalli et al., 2012). The main advantage of IPA is related with the formation of acetone as the only by-product, which can be easily removed from the reaction mixture through distillation. On the contrary, acetylation reactions performed with Ac_2_O lead to the formation of acetic acid, which must be removed through acid–base extractions. In one of the first reported examples concerning IPA application in acetylation reactions, 20% mol K_2_CO_3_ was the catalyst (Barry et al., 1988). Although the reaction proceeded with an almost quantitative yield at room temperature, the environmental impact of the process was negatively influenced by the strong basicity and high amount of the base. In fact, after reaction, high volumes of water were possibly needed to neutralize the K_2_CO_3_ solution, thus making the process less sustainable and raising disposal budgets. Such treatments are absent in VOSO_4_-catalyzed reactions. Thus, 1% of vanadyl sulfate has been used as the catalyst in the *O*-acetylation of alcohols and phenols, using isopropenyl acetate as both reagent and solvent in a stoichiometric amount (Figure 3). However, at room temperature, *O-*acetylation did not occur with satisfactory yields; therefore, reactions have been carried out at 60°C. Remarkably, thymol and carvacrol acetylation were accomplished with ca. 75% yield, which is in line with the results achieved with Ac_2_O; conversely, octyl, cyclohexyl, and benzyl acetate were obtained in lower amounts, and the reaction performed on aryl and alkyl thiols led to poor yields and lacked in selectivity (Supplementary Table S7).

## Conclusion

In this study, a sustainable procedure to carry out acylation reactions has been proposed. Thymol has been adopted as model substrate, as its ester derivatives are particularly known for their biological properties. Results showed that it is possible to successfully perform thymol acetylation using a stoichiometric amount of acetic anhydride in solvent-free conditions. The activation of acetic anhydride is promoted by the addition of 1% of VOSO_4_. Here, thymyl acetate can be isolated in 80% yield in 24 h at room temperature. The optimized process shows an E-factor decisively lower than that of the classical acylation reactions, which requires the use of a large excess of acetic anhydride and organic solvents. The advantages of the process have been confirmed by preliminary scale-up studies, which showed that the 50 g scale reaction proceeds with an almost quantitative substrate conversion. Furthermore, the optimized conditions can be efficiently adopted for the synthesis of thymyl esters using different anhydrides, as well as for the acylation of other phenols, alkyl alcohols, and alkyl and aryl thiols.

Interestingly, a further improvement of the sustainability of the process was achieved by carrying out the reaction with a stoichiometric amount of isopropenyl acetate, which is a “greener” alternative to acetic anhydride, as the only by-product is acetone, which is easily removed from the reaction mixture through distillation. Again, good results in phenol acetylation have been obtained at 60°C, although the reaction is worth to be further optimized for alkyl alcohols and thiols.

## Data Availability

The original contributions presented in the study are included in the article/Supplementary Material, further inquiries can be directed to the corresponding author.
