# Supplementary material for: A Stoichiometric Solvent-Free Protocol for Acetylation Reactions
Source: Front Chem. 2022 Mar 9;10:842190. doi: 10.3389/fchem.2022.842190 (PMC8959667; doi:10.3389/fchem.2022.842190)
Supplement: Supplementary file 1 [file DataSheet1.pdf]

Supplementary Material

Index

1. Product characterization 2

2. Tables 5

3. ^{1}H-NMR and ^{13}C-NMR Spectra 12

4. Mass Spectrum 25

5. GC-MS Calibration Curves 28

6. References 35

1. Products characterization

Thymyl Acetate **(3)**[1]

Colorless oil. Isolated yields are reported in Table 1. ^{1}H-NMR in $CDCl_3$: δ 7.19 (d, 1H), δ 7.02 (d, 1H), δ 6.80 (s, 1H), δ 3.20-2.91 (m, 1H), δ 2.31 (s, 6H), δ 1.19 (d, 6H). ^{13}C-NMR in $CDCl_3$: δ 169.99, 148.05, 137.18, 136.75, 127.35, 126.65, 122.96, 27.31, 23.27, 23.15, 21.12, 20.99. MS (EI): *m/z* (%) = 192 (11) $[M]^+$, 193 (2) $[M + 1]^+$, 150 (41) $[M + 1 – C(O)CH_3]^+$, 135 (100) $[150 – CH_3]^+$.

Synthesis of Thymyl propionate **(3a)**[2]

Colorless oil. Isolated yield after 2 hours = 63% (865 mg, 4.2 mmol); isolated yield after 24 hours = 78% (1.06 g, 5.1 mmol). ^{1}H-NMR in $CDCl_3$: δ 7.19 (d, 1H), δ 7.01 (d, 1H), δ 6.80 (s, 1H), δ 2.99-2.93 (m, 1H), δ 2.62-2.59 (q, 2H), δ 2.31 (s, 3H), δ 1.29 (t, 3H), δ 1.19 (d, 6H). ^{13}C-NMR in $CDCl_3$: δ 173.34, 148.14, 137.17, 136.70, 127.20, 126.56, 122.98, 27.94, 27.29, 23,23, 23,12, 20.99, 9.38. MS (EI): *m/z* (%) = 206 (7) $[M]^+$, 207 (1) $[M + 1]^+$, 150 (42) $[M + 1 – C(O)CH_2CH_3]^+$, 135 (100) $[150 – CH_3]^+$.

Synthesis of Thymyl butanoate **(3b)**[1]

Colorless oil. Isolated yield after 2 hours = 69% (1.01 g, 4,5 mmol); isolated yield after 24 hours = 78% (1.13 g, 5.1 mmol). ^{1}H-NMR in $CDCl_3$: δ 7.19 (d, 1H), δ 7.01 (d, 1H), δ 6.79 (s, 1H), δ 2.99-2.93 (m, 1H), δ 2.57-2.54 (t, 2H), δ 2.31 (s, 3H), δ 1.83-1.78 (m, 2H), δ 1.19 (d, 6H), δ 1.05 (t, 3H). ^{13}C-NMR in $CDCl_3$: δ 172.53, 148.11, 137.19, 136.69, 127.19, 126.55, 122.99, 36.45, 27.25, 23.25, 23.14, 20.99, 18.75, 13.89. MS (EI): *m/z* (%) = 220 (7) $[M]^+$, 221 (1) $[M + 1]^+$, 150 (52) $[M + 1 – C(O)CH_2CH_2CH_3]^+$, 135 (100) $[150 – CH_3]^+$.

Synthesis of Thymyl trimethylacetate **(3c)**

Colorless oil. Isolated yield after 2 hours = 31% (483 mg, 2.1 mmol); isolated yield after 24 hours = 40% (622 mg, 2.3 mmol). ^{1}H-NMR in $CDCl_3$: δ 7.18 (d, 1H), δ 7.00 (d, 1H), δ 6.76 (s, 1H), δ 2.99-2.93 (m, 1H), δ 2.30 (s, 3H), δ 1.37 (s, 9H), δ 1.18 (d, 6H). ^{13}C-NMR in $CDCl_3$: δ 177.35, 148.45, 137.19, 136.67, 127.01, 126.34, 122.85, 39.35, 27.46, 27.12, 23.15, 23.06, 21.00. MS (EI): *m/z* (%) = 234 (6) $[M]^+$, 235 (1) $[M + 1]^+$, 177 (1) $[M – C(CH_3)_3]^+$, 150 (22) $[M +1 – C(O)C(CH_3)_3]^+$, 135 (69) $[150 – CH_3]^+$, 57 (100) $[C(CH_3)_3]^+$.

Synthesis of Thymyl trifluoroacetate **(3d)**

Colorless oil. Isolated yield after 24 hours = 99% (1,60 g, 6,5 mmol). ^{1}H-NMR in $CDCl_3$: δ 7.28 (d, 1H), δ 7.15 (d, 1H), δ 6.94 (s, 1H), δ 3.01-2.95 (m, 1H), δ 2.37 (s, 3H), δ 1.24 (d, 6H). ^{13}C-NMR in $CDCl_3$: δ 156.53, 146.79, 137.48, 136.70, 128.74, 127.25, 121.70, 27.36, 23.04, 20.99. MS (EI): *m/z* (%) = 246 (54) $[M]^+$, 247 (7) $[M + 1]^+$, 231 (100) $[M - CH_3]^+$, 213 (48) $[M + 1 - CH_3F]^+$, 149 (?) $[M - C(O)CF_3]^+$, 115 (68).

Synthesis of Carvacryl Acetate **(5a)**[2]

Chromatography column: SiO_2, DCM: petroleum ether 1:1 *v:v*. Colorless oil. Isolated yield after 2 hours = 76% (970 g, 5 mmol); isolated yield after 24 hours = 79% (1 g, 5.2 mmol). ^{1}H-NMR in $CDCl_3$: δ 7.14 (d, 1H), δ 7.01 (d, 1H), δ 6.85 (s, 1H), δ 2.90-2.84 (m, 1H), δ 2.31 (s, 3H), δ 2.13 (s, 3H), δ 1.23 (d, 6H). ^{13}C-NMR in $CDCl_3$: δ 169.48, 149.45, 148.24, 131.06, 127.33, 124.34, 119.96, 33.72, 24.12, 24.01, 21.00, 15.88. MS (EI): *m/z* (%) = 192 (8) $[M]^+$, 193 (1) $[M + 1]^+$, 150 (62) $[M + 1 - C(O)CH_3]^+$, 135 (100) $[150 - CH_3]^+$.

Synthesis of Phenyl Acetate **(5b)**[3]

Chromatography column: SiO_2, DCM. Colorless oil. Isolated yield after 24 hours = 77% (1.1 g, 8.1 mmol). ^{1}H-NMR in $CDCl_3$: δ 7.37 (t, 2H), δ 7.22(t, 1H), δ 7.08 (d, 2H), δ 2.30 (s, 3H). ^{13}C-NMR in $CDCl_3$: δ 169.68, 150.92, 129.64, 126.03, 121.79, 21.32 MS (EI): *m/z* (%) = 136 (18) $[M]^+$, 94 (100) $[PhOH]^+$, 43 (25) $[C(O)CH_3]^+$.

Synthesis of Octyl Acetate **(5c)**[3]

Chromatography column: SiO_2, CH_3Cl. Colorless oil. Isolated yield after 24 hours = 72% (946 mg, 5,4 mmol). ^{1}H-NMR in $CDCl_3$: δ 4.05 (t, 2H), δ 2.04 (s, 3H), δ 1.63-1.59 (m, 2H), δ 1.33-1.26 (m, 10H), δ 0.88 (t, 3H). ^{13}C-NMR in $CDCl_3$: δ 171.51, 64.94, 32.06, 29.50, 29.46, 28.89, 26.20, 22.92, 21.28, 14.35. MS (EI): m/z (%) = 43 (100) $[C(O)CH_3]^+$.

Synthesis of Cyclohexyl Acetate **(5d)**[3]

Chromatography column: SiO_2, DCM. Colorless oil. Isolated yield after 24 hours = 61% (864 mg, 6 mmol). ^{1}H-NMR in $CDCl_3$: δ 4.75-4.71 (m, 1H), δ 2.02 (s, 3H), δ 1.85-1.83 (m, 2H), δ 1.73-1.70 (m, 2H), δ 1.54-1.53 (m, 1H), δ 1.42-1.32 (m, 4H), δ 1.26-1.21 (m, 1H). ^{13}C-NMR in $CDCl_3$: δ 170.87, 72.95, 31.93, 25.65, 24.08, 21.71. MS (EI): *m/z* (%) = 82 (64) $[M - CH_3C(O)OH]^+$, 67 (58) $[82 - CH_3]^+$, 43 (100) $[CH_3C(O)]^+$.

Synthesis of Benzyl Acetate **(5e)**[3]

Chromatography column: SiO_2, DCM. Colorless oil. Isolated yield after 2 hours = 76% (1.06 g, 7 mmol); isolated yield after 24 hours = 87% (1.2 g, 8 mmol). ^{1}H-NMR in $CDCl_3$: δ 7.36-7.31 (m, 5H), δ 5.11 (s, 2H), δ 2.10 (s, 3H). ^{13}C-NMR in $CDCl_3$: δ 171.03, 136.11, 128.72, 128.42, 128.40, 66.46, 21.16. MS (EI): *m/z* (%) = 150 (37) $[M]^+$, 108 (100) $[M – C(O)CH_3]^+$, 91 (66) $[108 – OH]^+$.

Synthesis of 4-methylphenyl thioacetate **(5f)**[3]

Chromatography column: SiO_2, DCM:petroleum ether 3:1 *v:v*. Colorless oil. Isolated yield after 24 hours = 95% Isolated Yield (1.26 g, 7.6 mmol) ^{1}H-NMR in $CDCl_3$: δ 7.29 (d, 2H), δ 7.22 (d, 2H), δ 2.40 (s, 3H), δ 2.37 (s, 3H). ^{13}C-NMR in $CDCl_3$: δ 194.84, 139.98, 134.68, 130.31, 124.72, 30.35, 21.59. MS (EI): *m/z* (%) =166 (10) $[M]^+$, 124 (100) $[4\text{-}CH_3PhSH]^+$, 91 (20) $[4\text{-}CH_3Ph]^+$, 43 (17) $[CH_3C(O)]^+$.

Synthesis of cyclohexyl thioacetate **(5g)**[4]

Chromatography column: SiO_2, DCM:petroleum ether 3:1 *v:v*. Colorless oil. Isolated yield after 24 hours = 82% (1.12 g, 7 mmol). ^{1}H-NMR in $CDCl_3$: δ 3.50 (m, 1H), δ 2.29 (s, 3H), δ 1.91-1.89 (m, 2H), δ 1.70-1.68 (m, 2H), δ 1.59-1.57 (m, 1H), δ 1.45-1.37 (m, 4H), δ 1.28-1.25 (m, 1H). ^{13}C-NMR in $CDCl_3$: δ 195.95, 42.61, 33.18, 30.95, 26.09, 25.72. MS (EI): *m/z* (%) = 158 (10) $[M]^+$, 82 (100) $[M – CH_3C(O)SH]^+$, 43 (70) $[CH_3C(O)]^+$.

Synthesis of octyl thioacetate **(5h)**[3]

Chromatography column: SiO_2, DCM:petroleum ether 3:1 *v:v*. Colorless oil. Isolated yield after 24 hours = 50% (642 mg, 3.4 mmol). ^{1}H-NMR in $CDCl_3$: δ 2.87-2.85 (t, 2H), δ 2.32 (s, 3H), δ 1.58-1.53 (q, 2H), δ 1.36-1.32 (m, 2H), δ 1.30-1.24 (m, 8H), δ 0.88-0.86 (t, 3H). ^{13}C-NMR in $CDCl_3$: δ 196.21, 31.98, 30.81, 29.70, 29.35, 29.34, 29.27, 29.02, 22.83, 14.27. MS (EI): *m/z* (%) = 145 (16) $[M – CH_3C(O)]^+$, 112 (9) $[M – CH_3C(O)SH]^+$, 43 (100) $[CH_3C(O)]^+$.

2. Tables

Table S1. Thymol esterification reactions. Reaction conditions: thymol = 6.7 mmol, $VOSO_4$ = 1%, anhydride = 1eq, T = r.t.

Entry	Anhydride (1 eq)	Product	Time (h)	GC-MS Yield (%)	Isolated Yield (%)
1	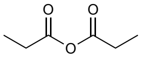	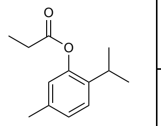	2	74	73
2			24	80	78
3	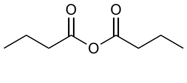	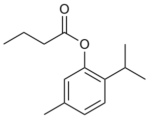	2	67	69
4			24	80	78
5	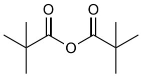	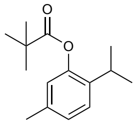	2	30	31
6			24	40	40
7	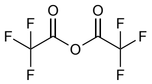	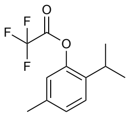	2	92	90
8			24	97	95

Table S2. Thymol acetylation reaction; blank experiments for different anhydrides. Reaction conditions: thymol = 6,7 mmol, Anhydride = 1eq, T = r.t.

Entry	Anhydride (1 eq)	Product	Time (h)	GC-MS Yield (%)
1	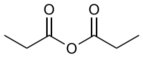	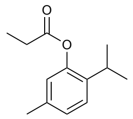	2	6
2			24	6,8
3	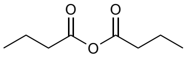	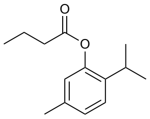	2	6
4			24	6,5
5	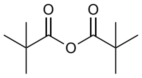	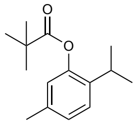	2	2
6			24	2,4
7	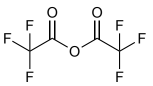	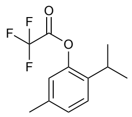	2	10
8			24	11

Table S3. Vanadium catalyzed acetylation of alcohols and phenol; investigation on substrate scope. Reaction conditions: substrate = 1g, acetic anhydride = 1 eq, $VOSO_4$ = 1%, T = r.t.

Entry	Substrate	Product	Time (h)	GC-MS Yield (%)	Isolated Yield (%)
1	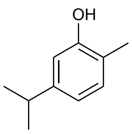	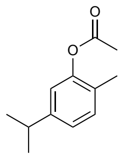	2	78	76
2			24	81	79
3	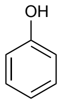	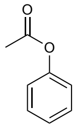	24	89	77
4	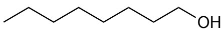	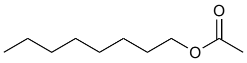	24	75	72
5	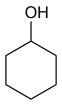	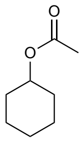	24	68	61
6	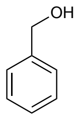	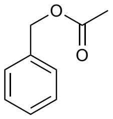	2	79	76
7			24	88	86

Table S4. Vanadium catalyzed acetylation of thiols; investigation on substrate scope. Reaction conditions: substrate = 1g, acetic anhydride = 1 eq, $VOSO_4$ = 1%, T = 60°C, t = 24 h.

Entry	Substrate	Product	GC-MS Yield (%)	Isolated Yield (%)
1	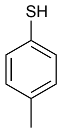	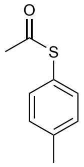	96	95
2	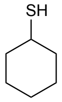	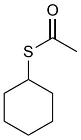	90	82
3	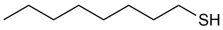	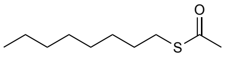	45	50

Table S5. *O* -acetylation reaction; blank experiments. Reaction conditions: substrate = 1 g, acetic anhydride = 1 eq, T= r. t., t = 24 h

Entry	Substrate	Product	GC-MS Yield (%)
1	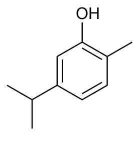	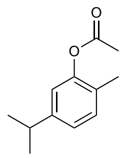	7
2	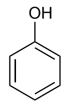	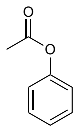	5
3	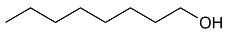	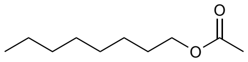	4
4	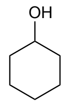	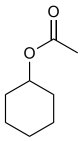	3
5	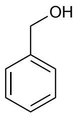	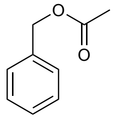	5

Table S6. *S*-acetylation reaction; blank experiments. Reaction conditions: substrate = 1 g, acetic anhydride = 1 eq, T = 60 °C, t = 24 h.

Entry	Substrate	Product	GC-MS Yield (%)
1	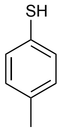	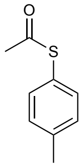	8
2	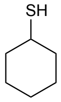	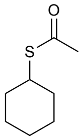	3
3	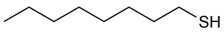	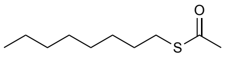	7

Table S7. Vanadium catalyzed acetylation of thymol, alcohols; investigation on substrate scope using isopropenyl acetate. Reaction condition: substrate = 1g, isopropenyl acetate = 1 eq, $VOSO_4$ = 1%, T = 60°C.

Entry	Substrate	Product	Time (h)	GC-MS Yield (%)
1	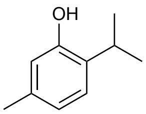	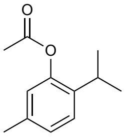	2	58
2			24	76
3	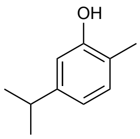	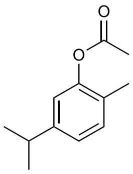	2	56
4			24	75
3	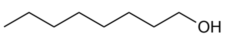	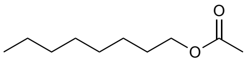	24	26
4	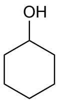	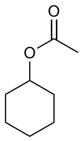	24	25
5	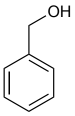	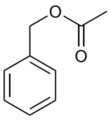	24	43
6	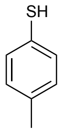	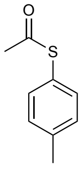	24	n. a.
7	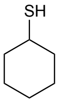	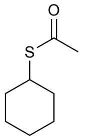	24	n. a.
8	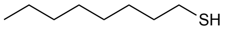	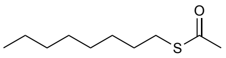	24	n. a.

3. ^{1}H-NMR and ^{13}C-NMR Spectra.

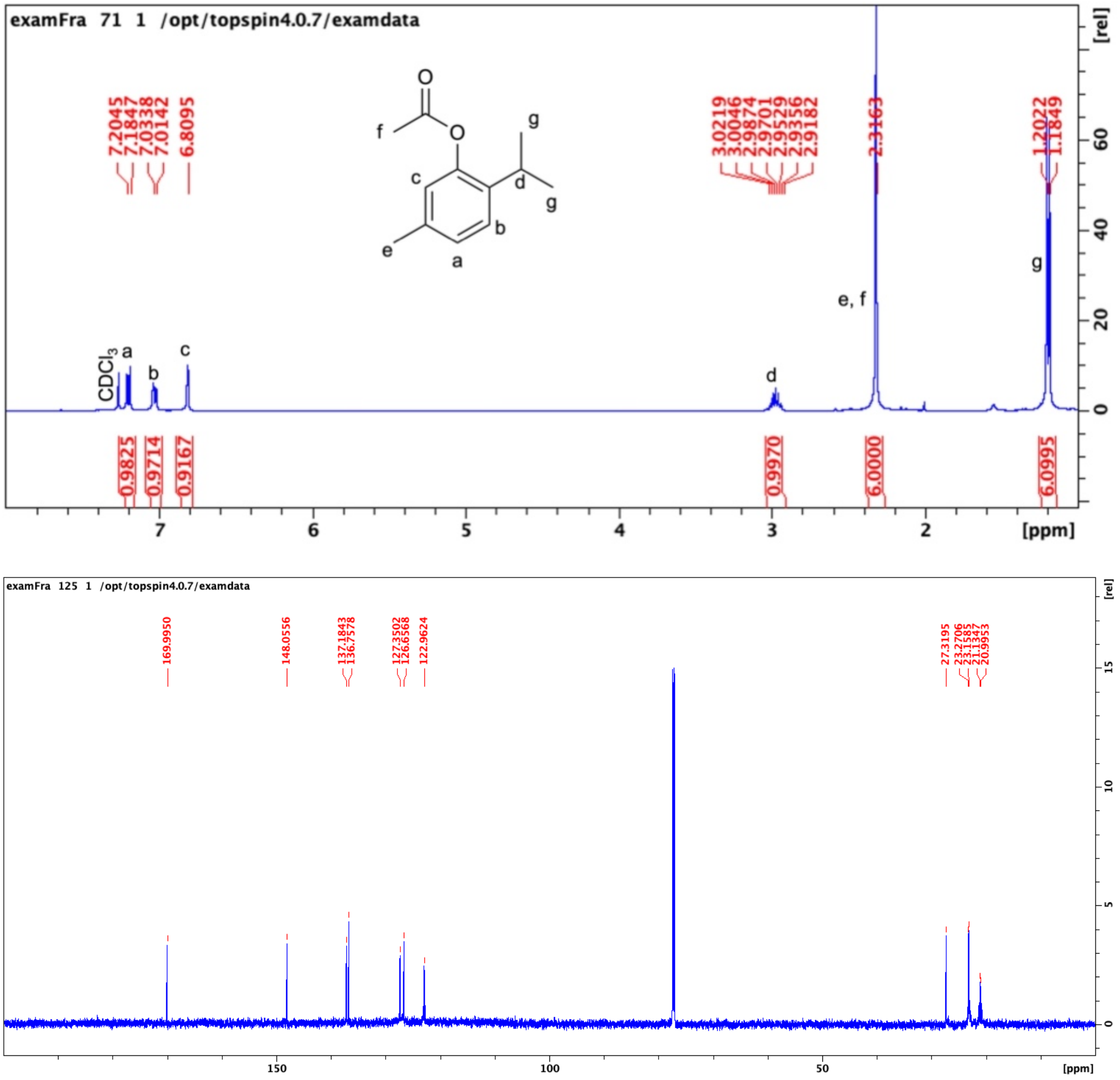

Supplementary Figure 1. ^{1}H-NMR (top) and ^{13}C-NMR (bottom) spectra of **3** in $CDCl_3$ (700 MHz).

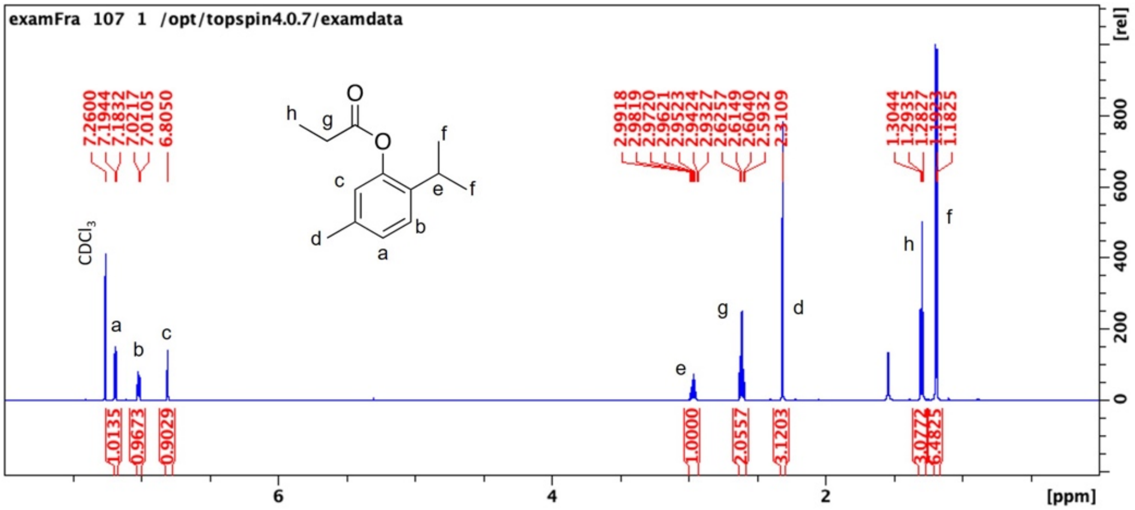

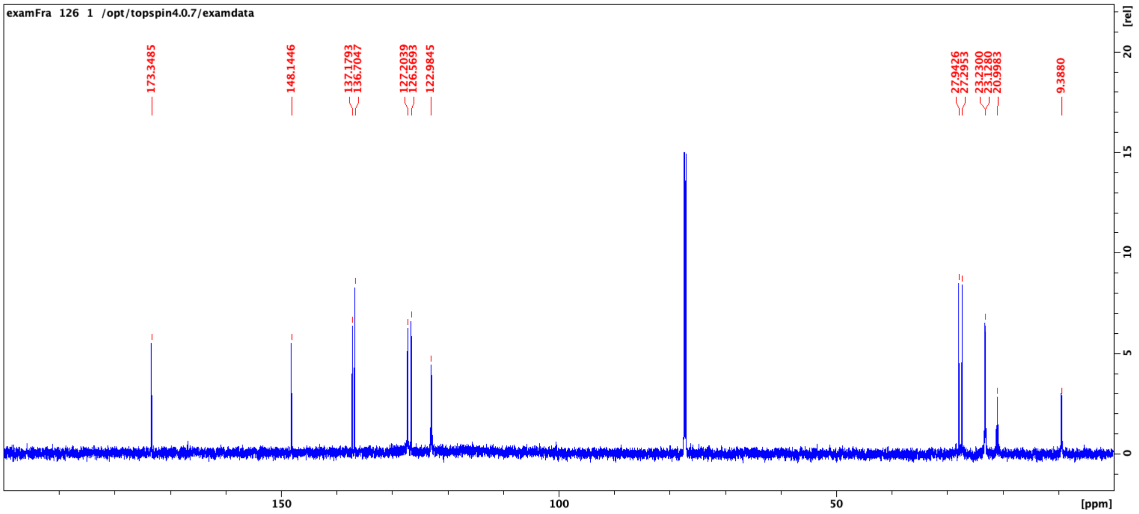

Supplementary Figure 2. ^{1}H-NMR (top) and ^{13}C-NMR (bottom) spectra of **3a** in $CDCl_3$ (700 MHz).

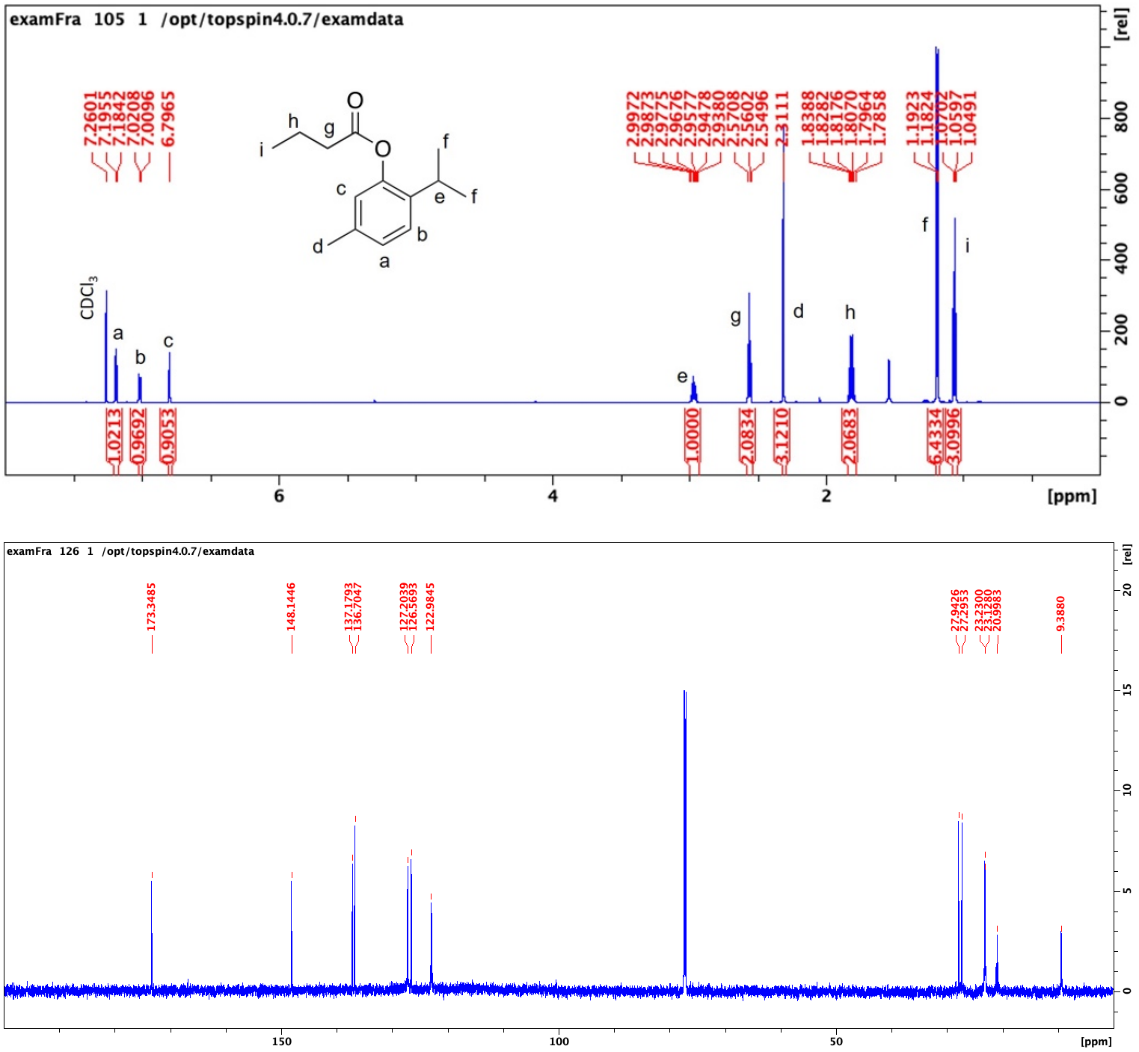

Supplementary Figure 3. ^{1}H-NMR (top) and ^{13}C-NMR (bottom) spectra of **3b** in $CDCl_3$ (700 MHz).

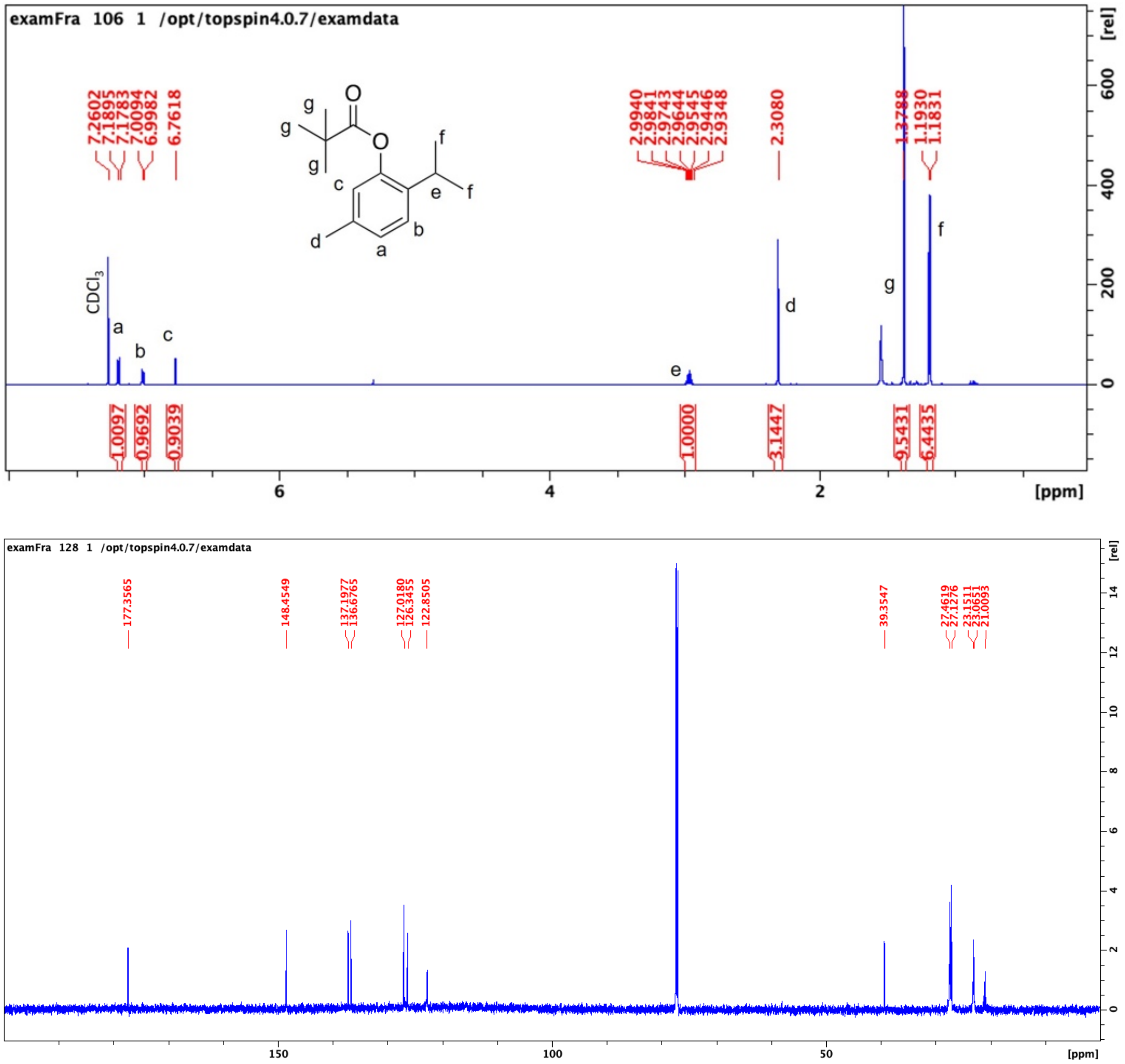

Supplementary Figure 4. ^{1}H-NMR (top) and ^{13}C-NMR (bottom) spectra of **3c** in $CDCl_3$ (700 MHz).

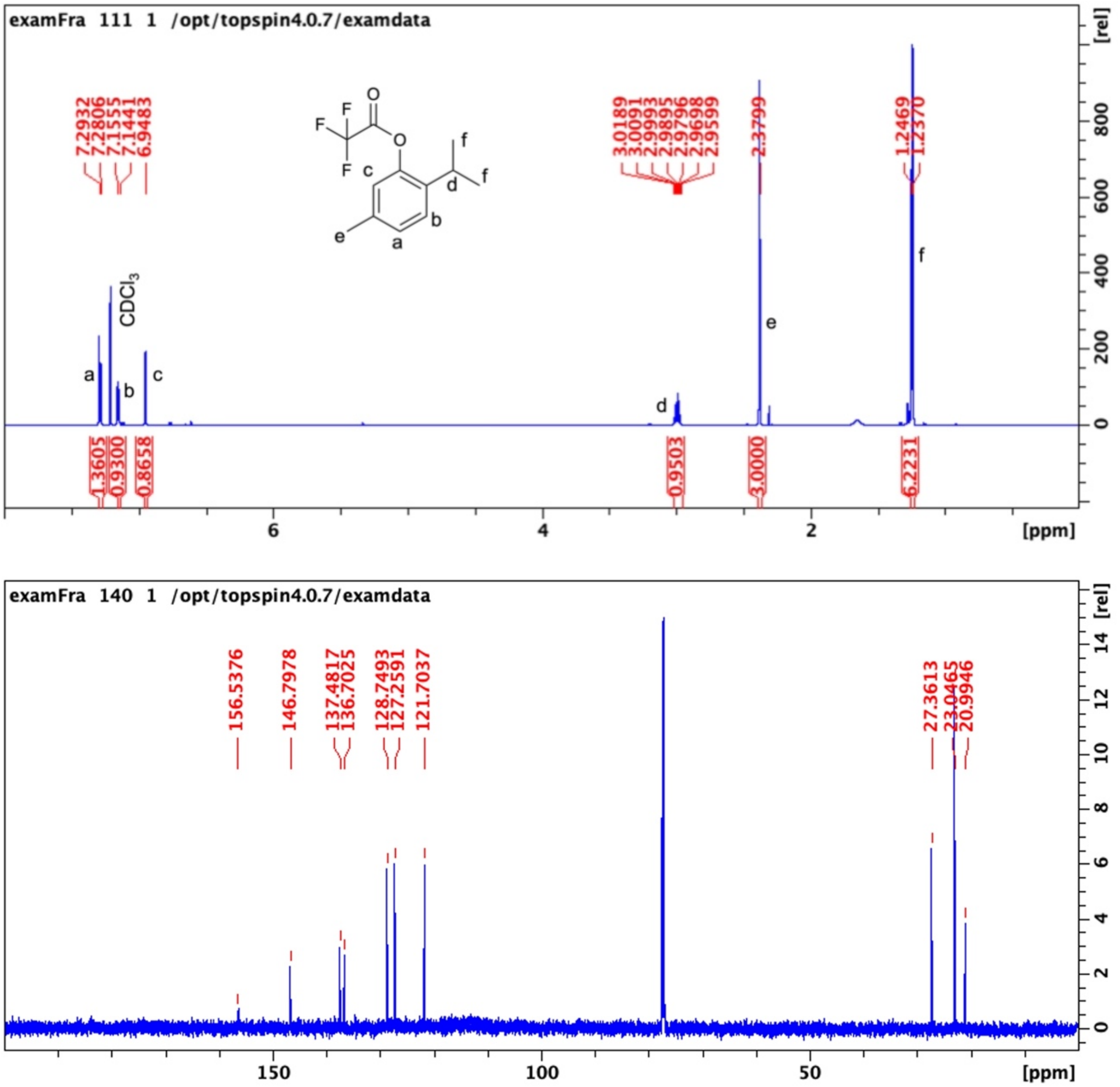

Supplementary Figure 5. ^{1}H-NMR (top) and ^{13}C-NMR (bottom) spectra of **3d** in $CDCl_3$ (700 MHz).

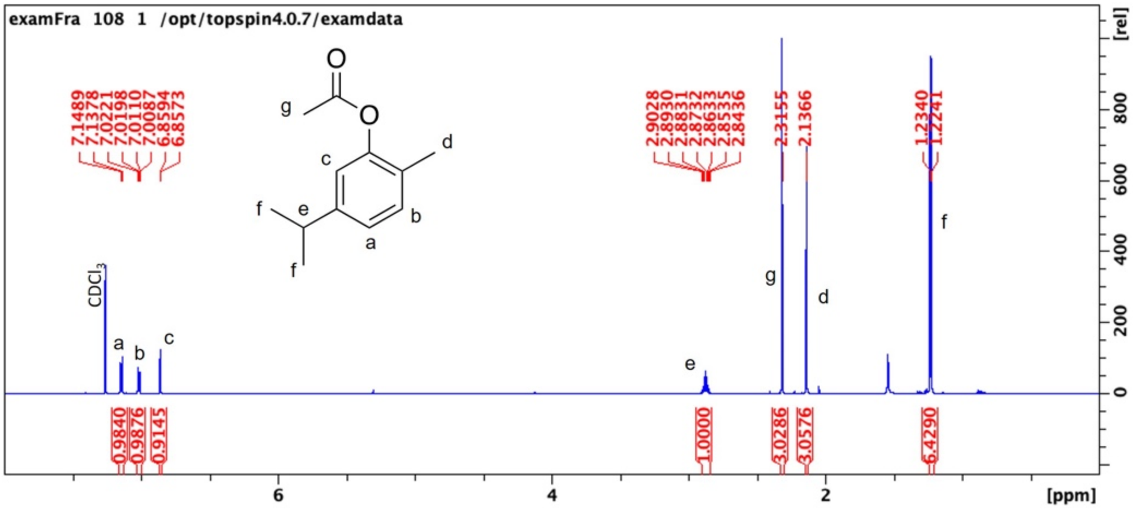

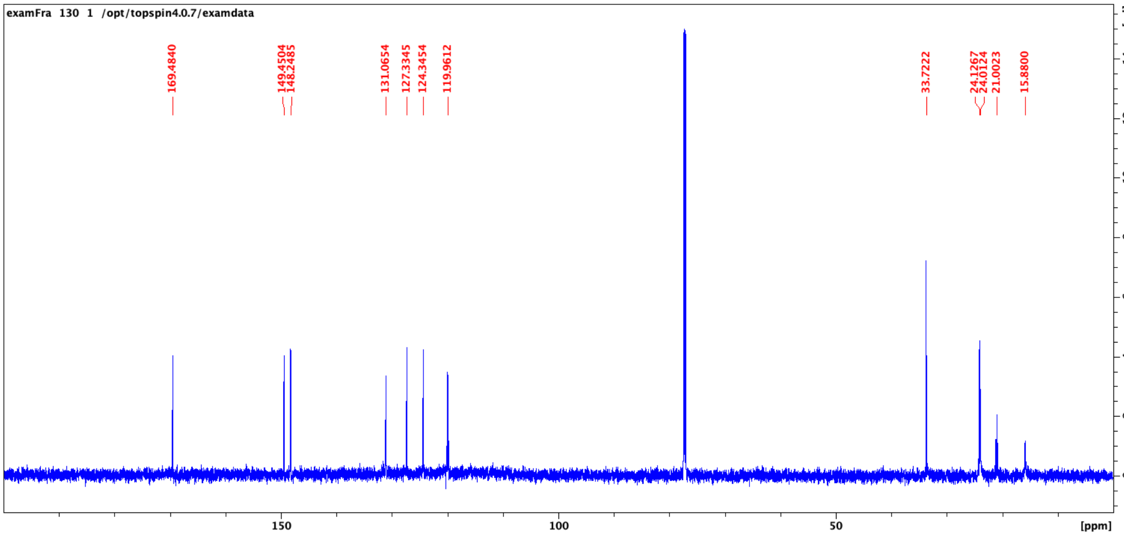

Supplementary Figure 6. ^{1}H-NMR (top) and ^{13}C-NMR (bottom) spectra of **5a** in $CDCl_3$ (700 MHz).

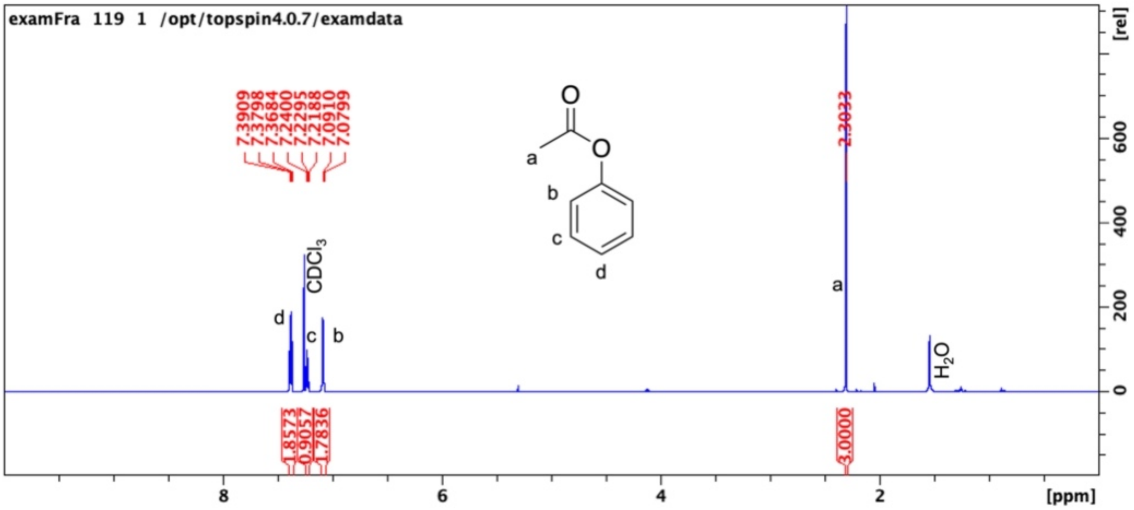

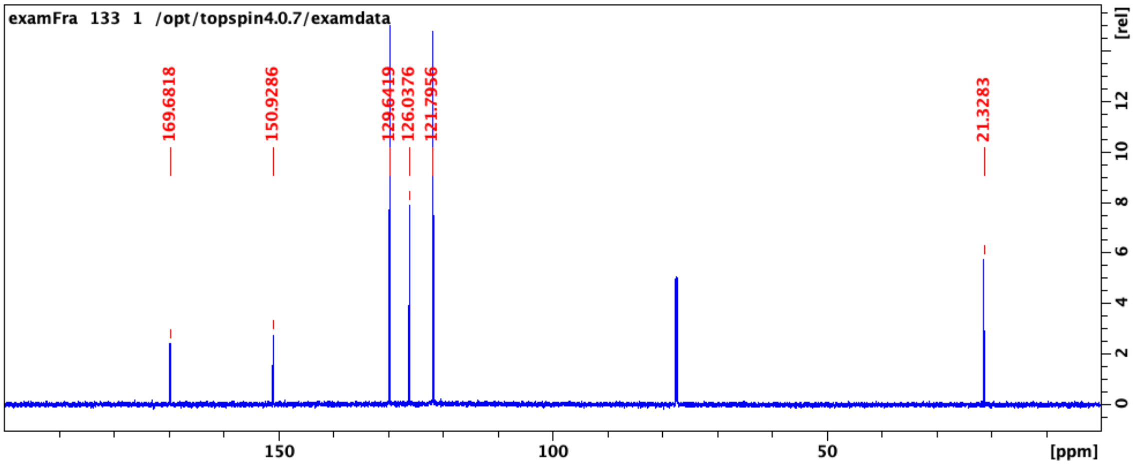

Supplementary Figure 7. ^{1}H-NMR (top) and ^{13}C-NMR (bottom) spectra of **5b** in $CDCl_3$ (700 MHz).

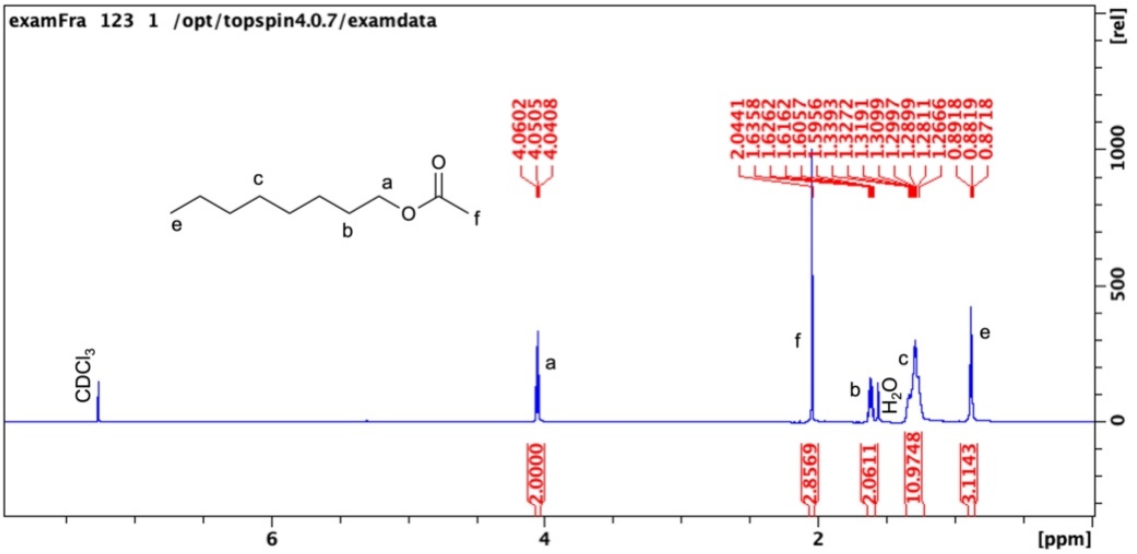

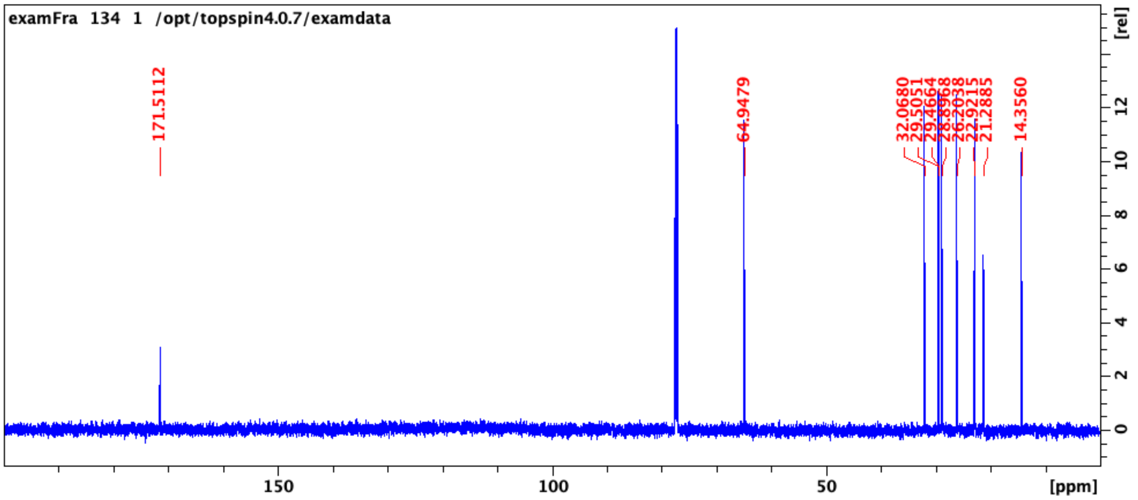

Supplementary Figure 8. ^{1}H-NMR (top) and ^{13}C-NMR (bottom) spectra of **5c** in $CDCl_3$ (700 MHz).

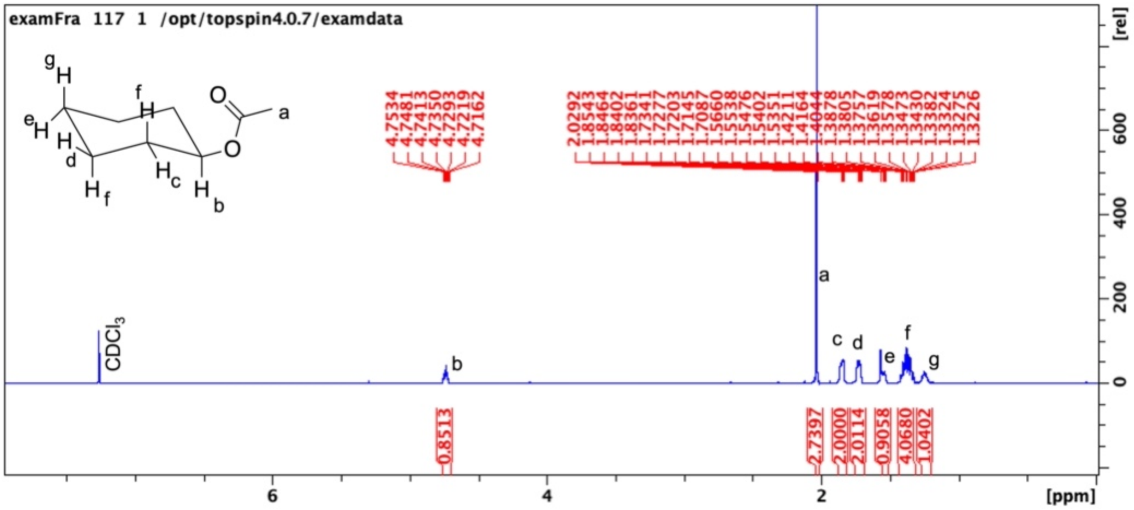

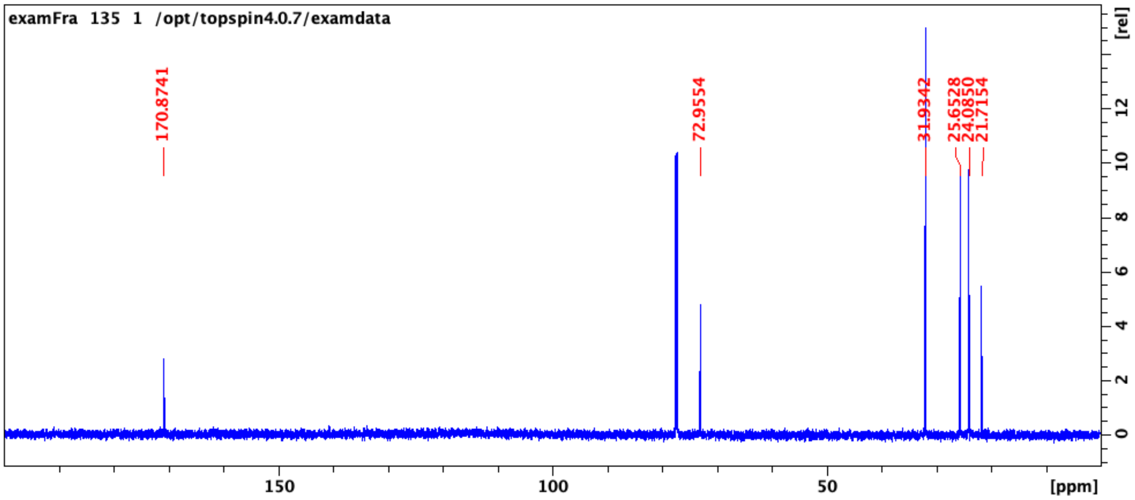

Supplementary Figure 9. ^{1}H-NMR (top) and ^{13}C-NMR (bottom) spectra of **5d** in $CDCl_3$ (700 MHz).

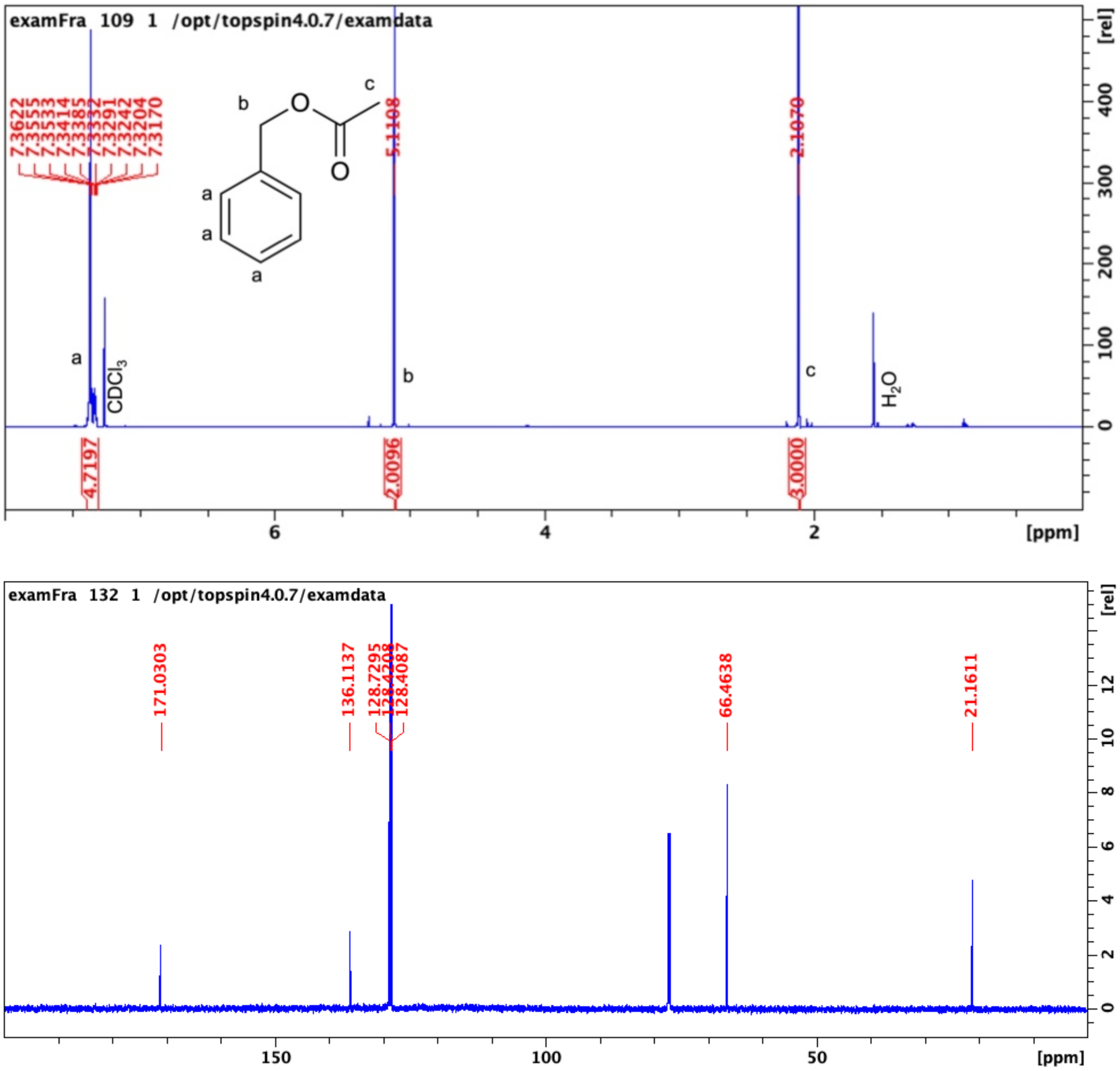

Supplementary Figure 10. ^{1}H-NMR (top) and ^{13}C-NMR (bottom) spectra of **5e** in $CDCl_3$ (700 MHz).

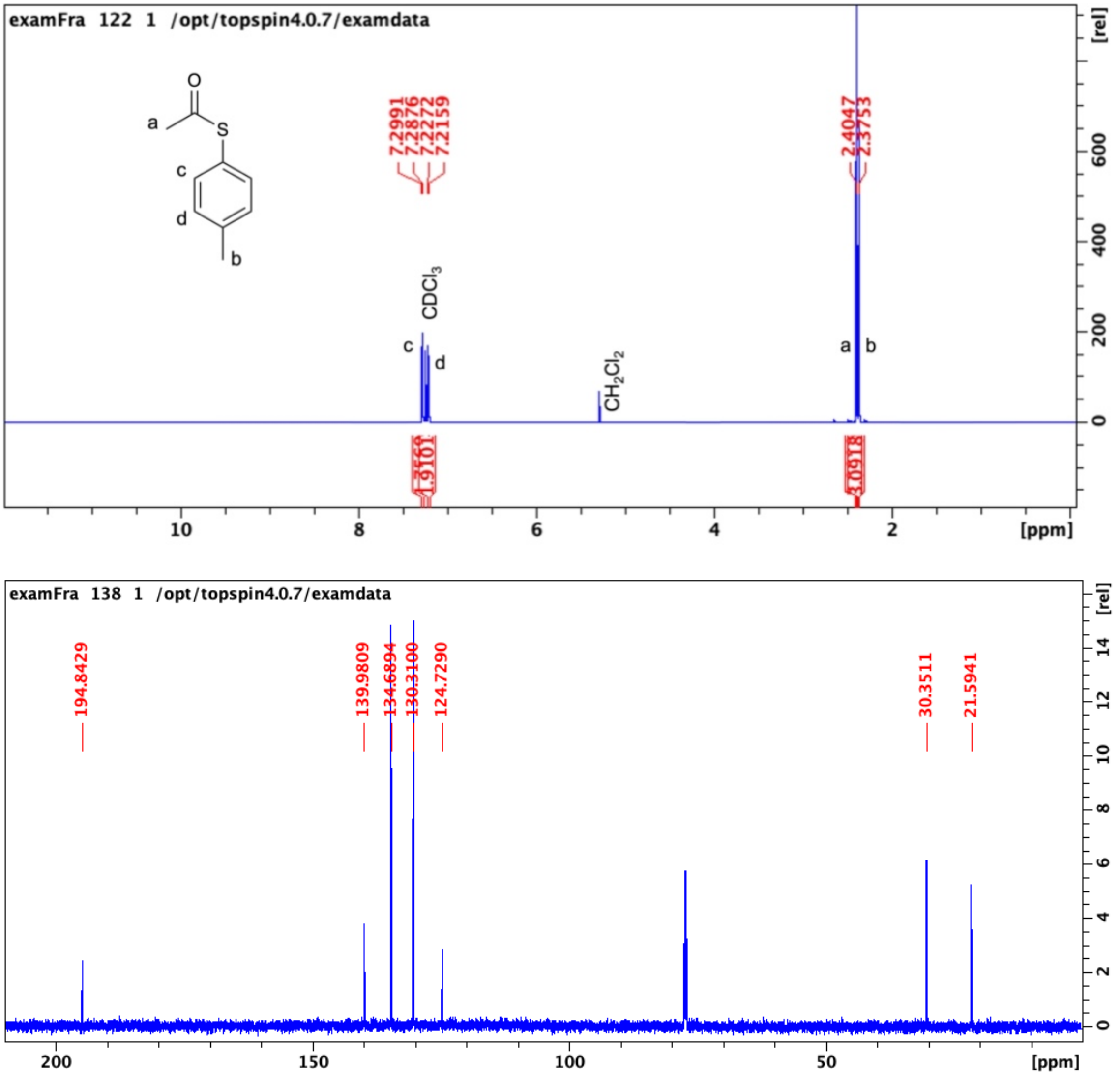

Supplementary Figure 11. ^{1}H-NMR (top) and ^{13}C-NMR (bottom) spectra of **5f** in $CDCl_3$ (700 MHz).

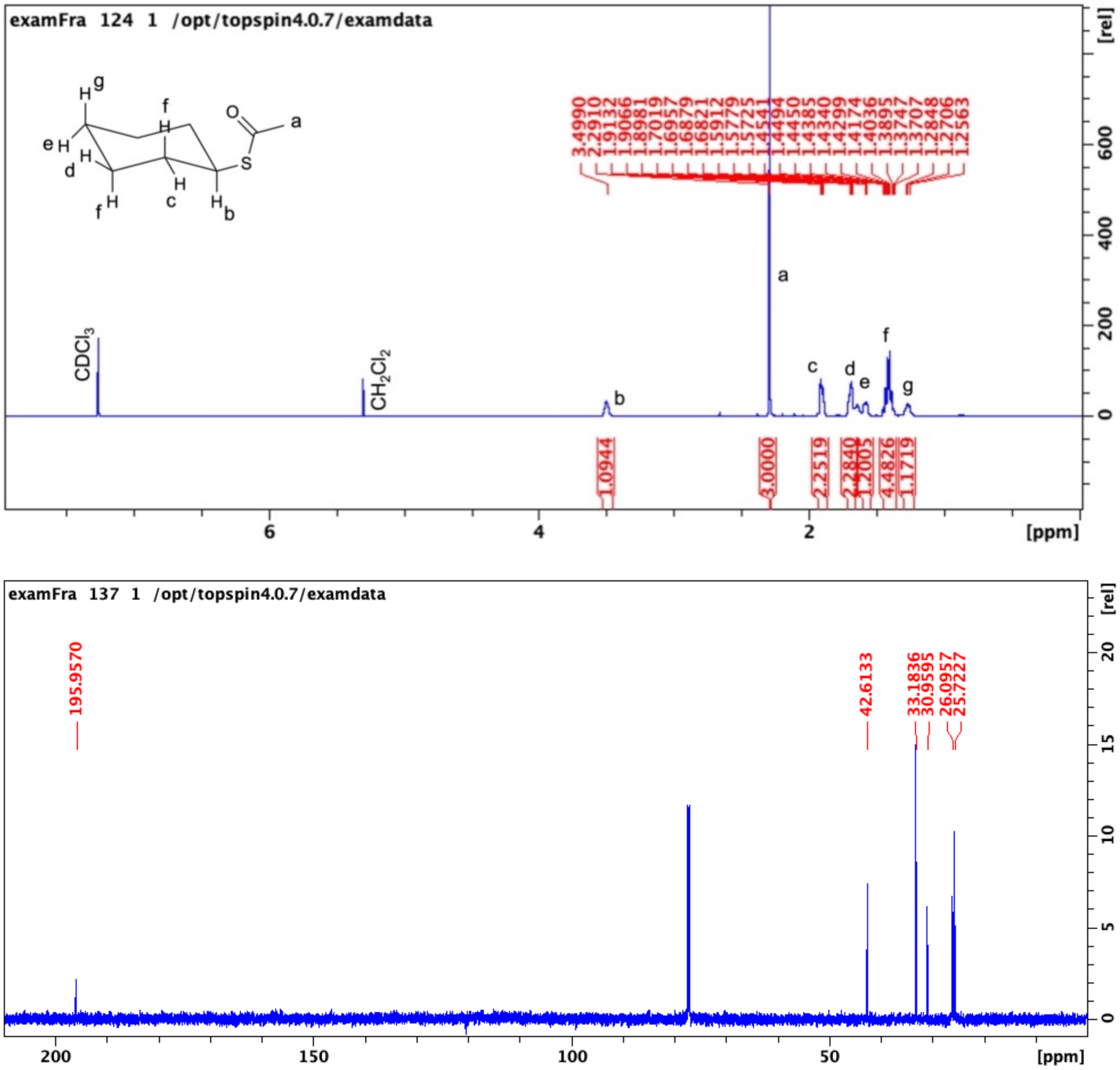

Supplementary Figure 12. ^{1}H-NMR (top) and ^{13}C-NMR (bottom) spectra of **5g** in $CDCl_3$ (700 MHz).

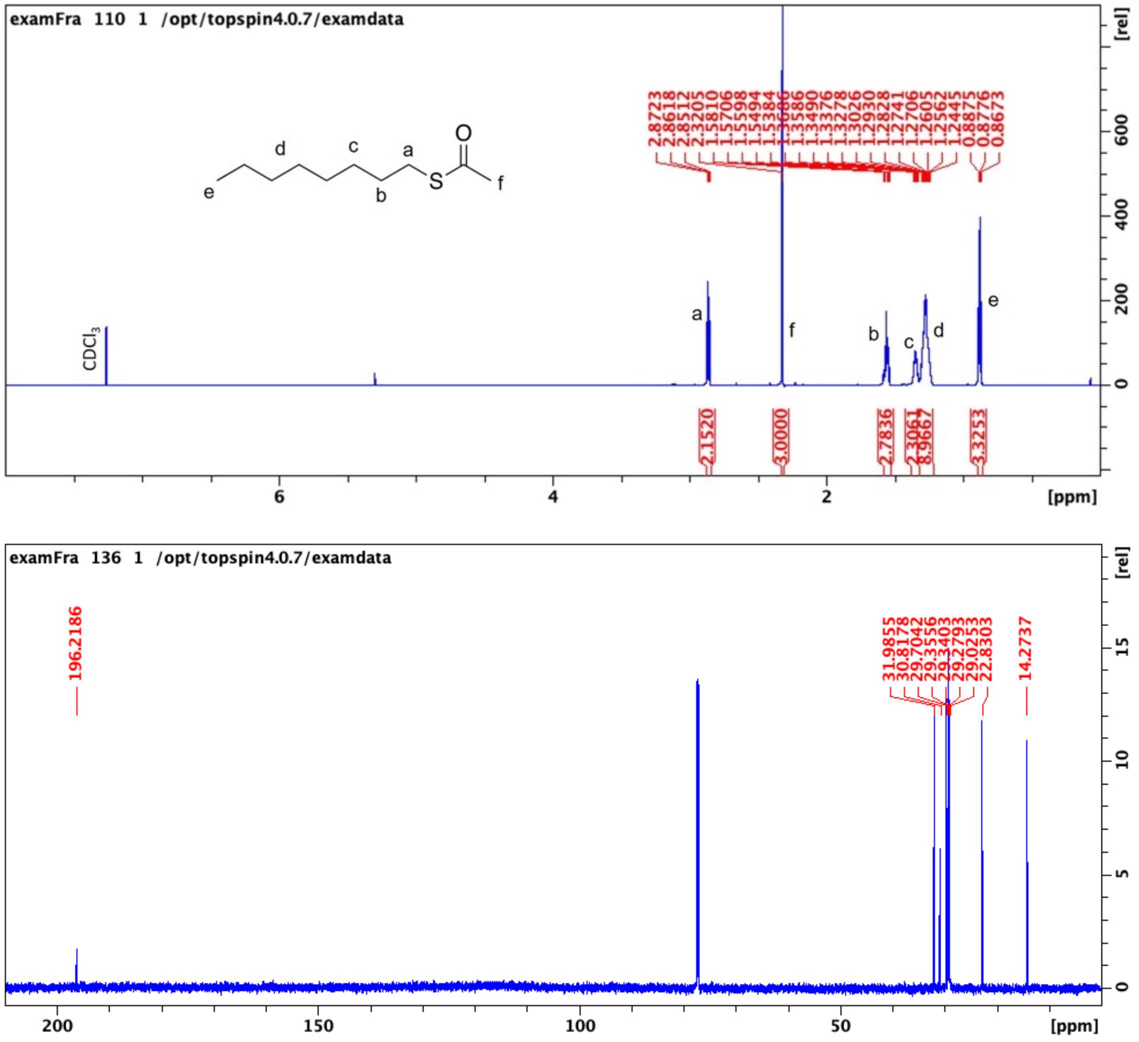

Supplementary Figure 13. ^{1}H-NMR (top) and ^{13}C-NMR (bottom) spectra of **5h** in $CDCl_3$ (700 MHz).

4. Mass Spectrum

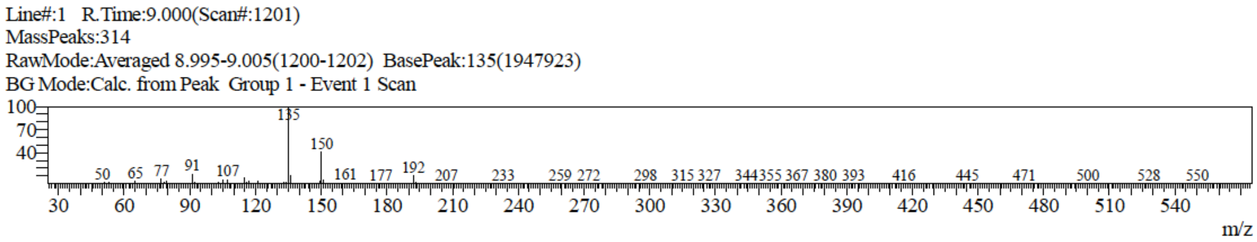

Supplementary Figure 14. *m/z* trace of **3.**

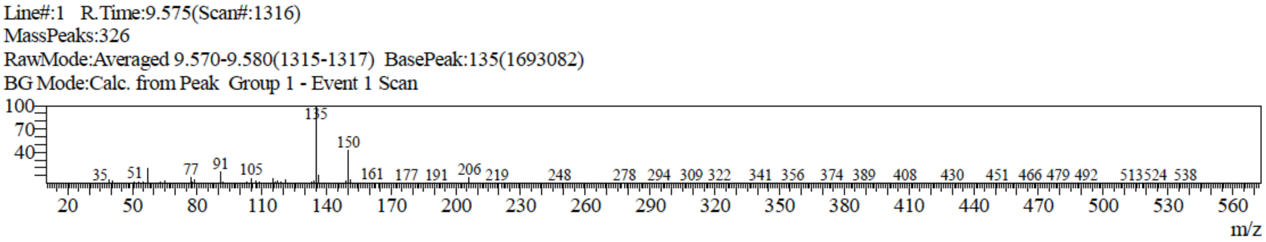

Supplementary Figure 15. *m/z* trace of **3a.**

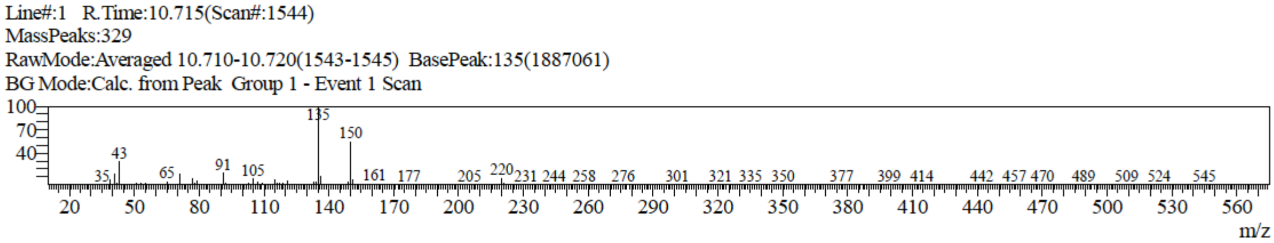

Supplementary Figure 16. *m/z* trace of **3b.**

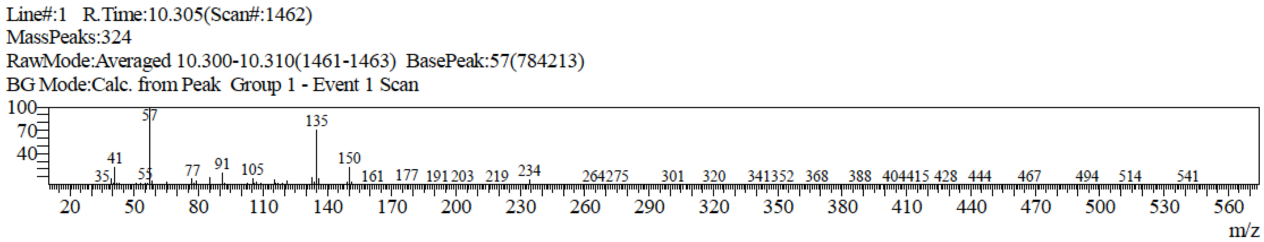

Supplementary Figure 17. *m/z* trace of **3c.**

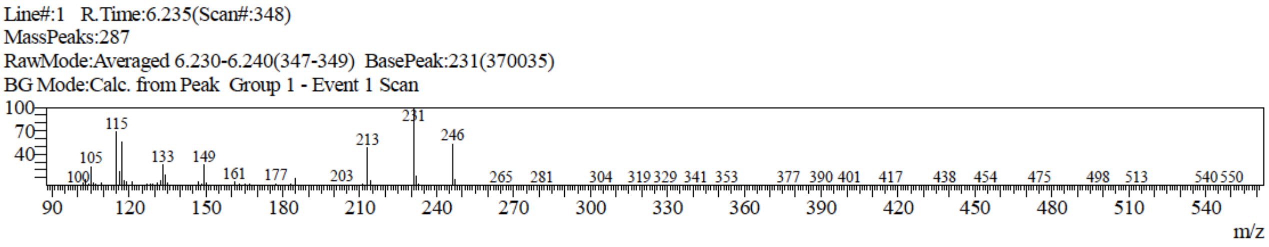

Supplementary Figure 18. *m/z* trace of **3d.**

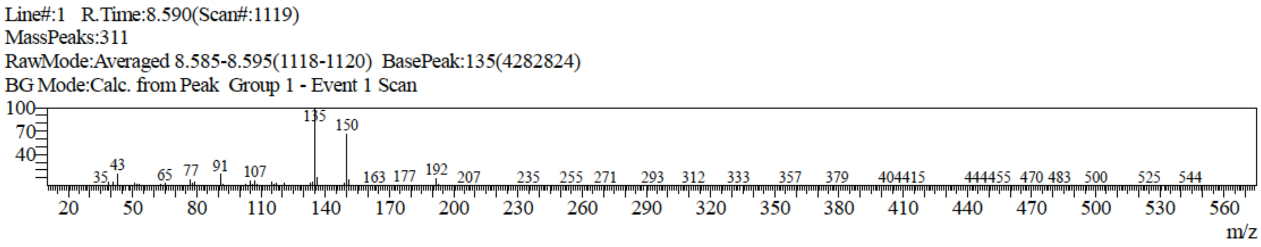

Supplementary Figure 19. *m/z* trace of **5a**

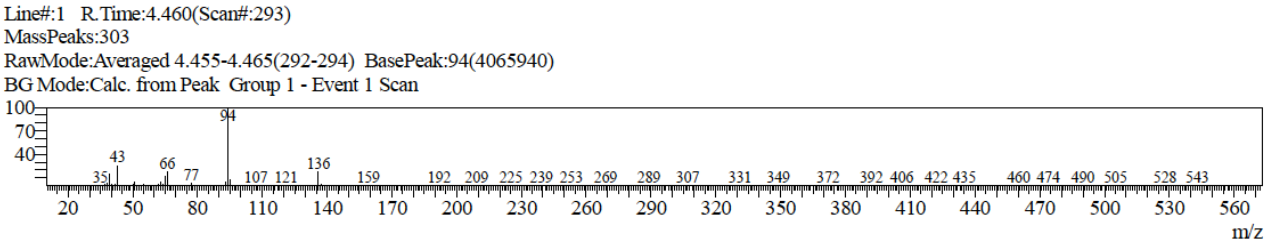

Supplementary Figure 20. *m/z* trace of **5b.**

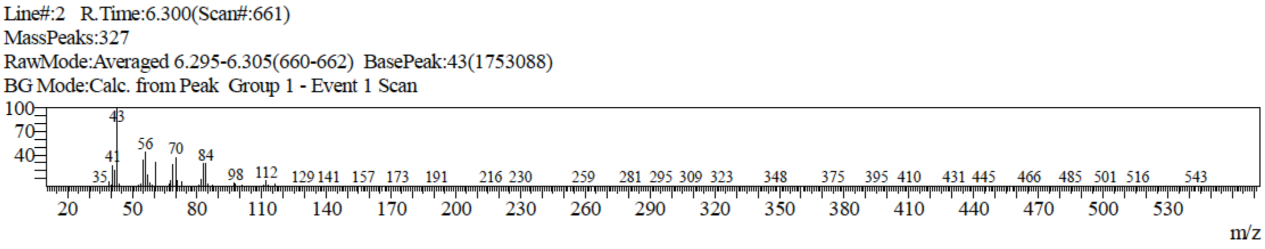

Supplementary Figure 21. m/z spectrum of **5c.**

Line#:1 R.Time:5.755(Scan#:552)
MassPeaks:315
RawMode:Averaged 5.750-5.760(551-553) BasePeak:43(2932299)
BG Mode:Calc. from Peak Group 1 - Event 1 Scan

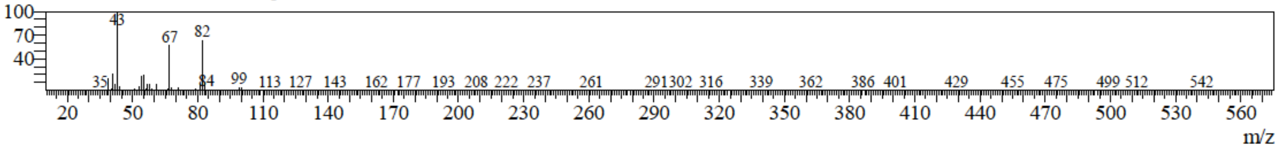

Supplementary Figure 22. m/z spectrum of **5d.**

Line#:1 R.Time:5.860(Scan#:573)
MassPeaks:343
RawMode:Averaged 5.855-5.865(572-574) BasePeak:108(3233688)
BG Mode:Calc. from Peak Group 1 - Event 1 Scan

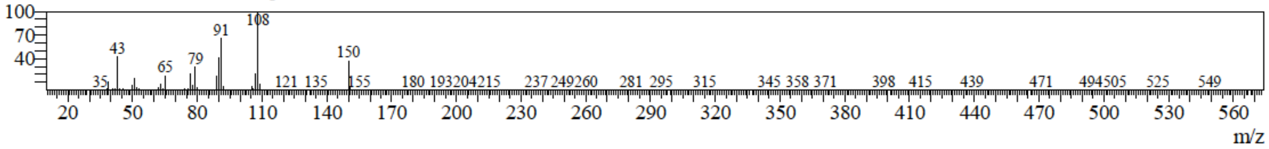

Supplementary Figure 23. m/z spectrum of **5e.**

Line#:1 R.Time:8.450(Scan#:1091)
MassPeaks:328
RawMode:Averaged 8.445-8.455(1090-1092) BasePeak:124(1732970)
BG Mode:Calc. from Peak Group 1 - Event 1 Scan

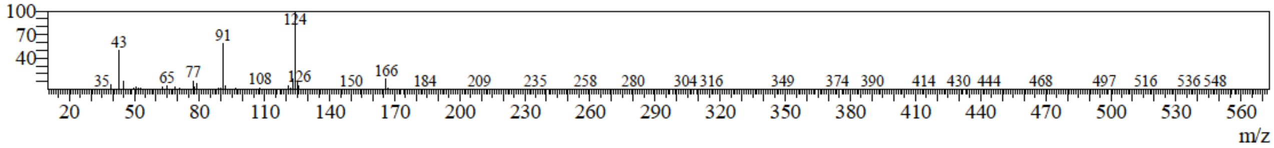

Supplementary Figure 24. m/z spectrum of **5f.**

Line#:1 R.Time:8.335(Scan#:1068)
MassPeaks:311
RawMode:Averaged 8.330-8.340(1067-1069) BasePeak:43(1456450)
BG Mode:Calc. from Peak Group 1 - Event 1 Scan

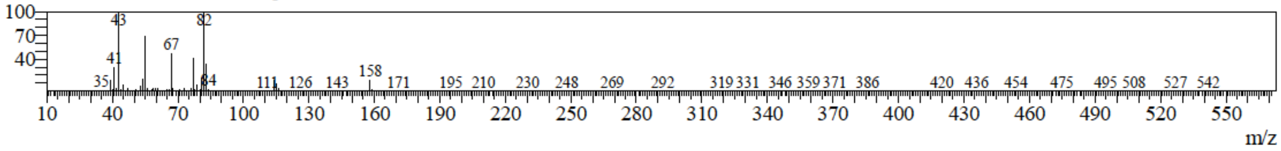

Supplementary Figure 25. m/z spectrum of **5g.**

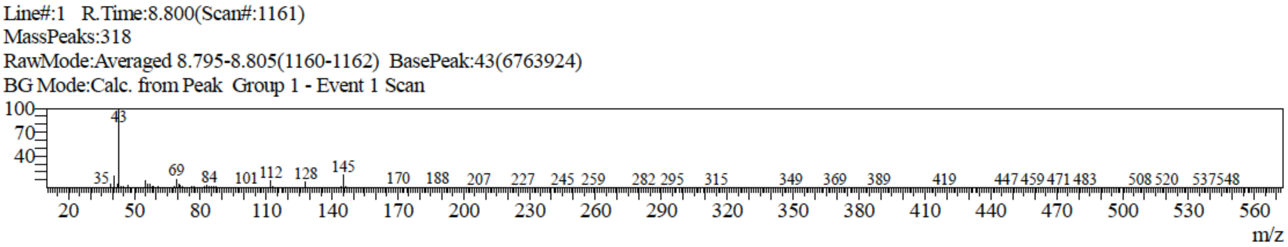

Supplementary Figure 26. m/z spectrum of **5h.**

5. GC-MS Calibration Curves

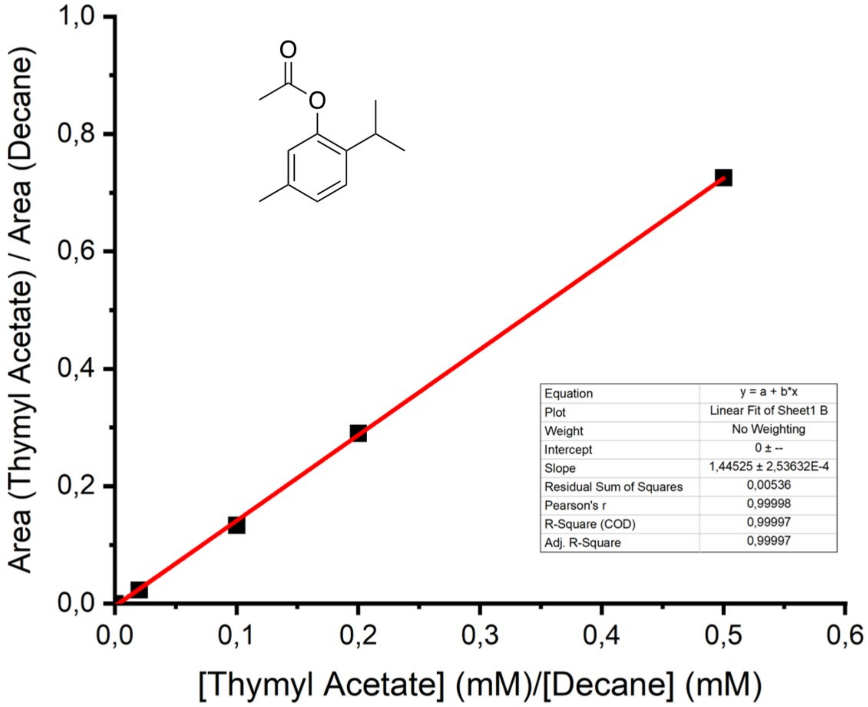

Supplementary Figure 27. 3 calibration curve using 10 mM decane as internal standard.

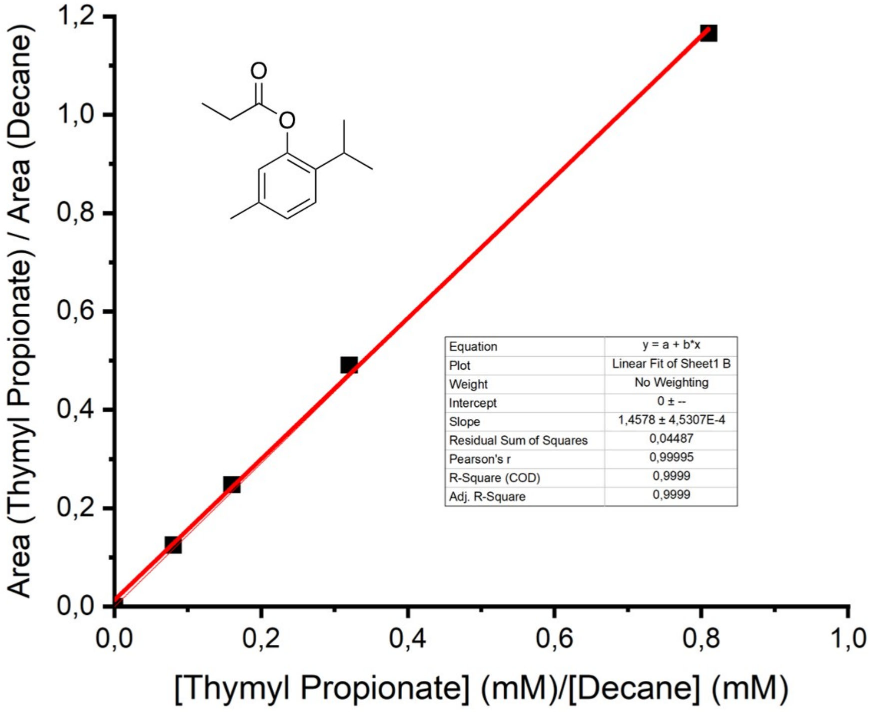

Supplementary Figure 28. 3a calibration curve using 10 mM decane as internal standard.

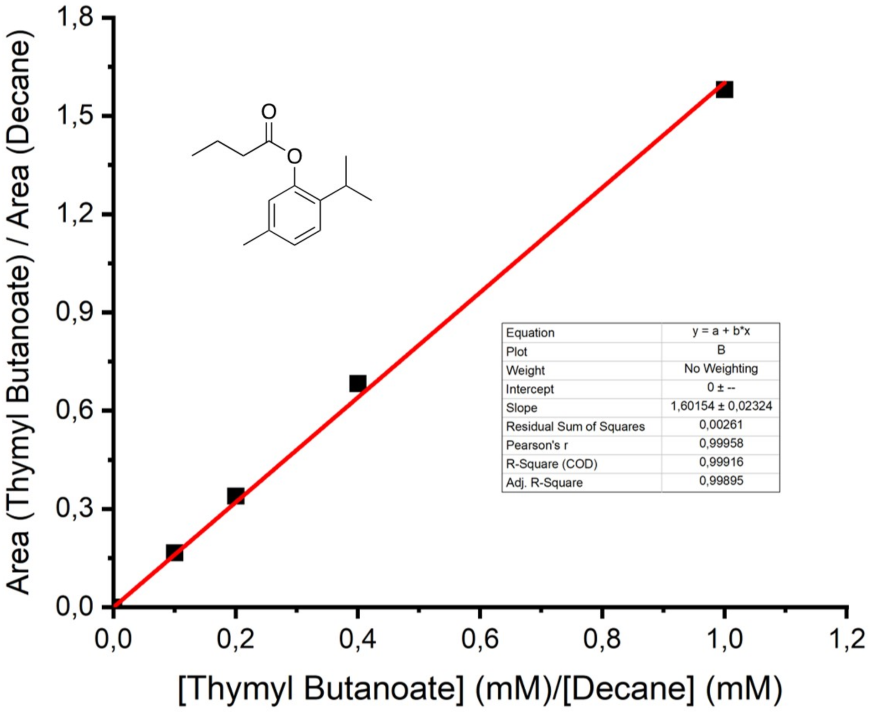

Supplementary Figure 29. 3b calibration curve using 10 mM decane as internal standard.

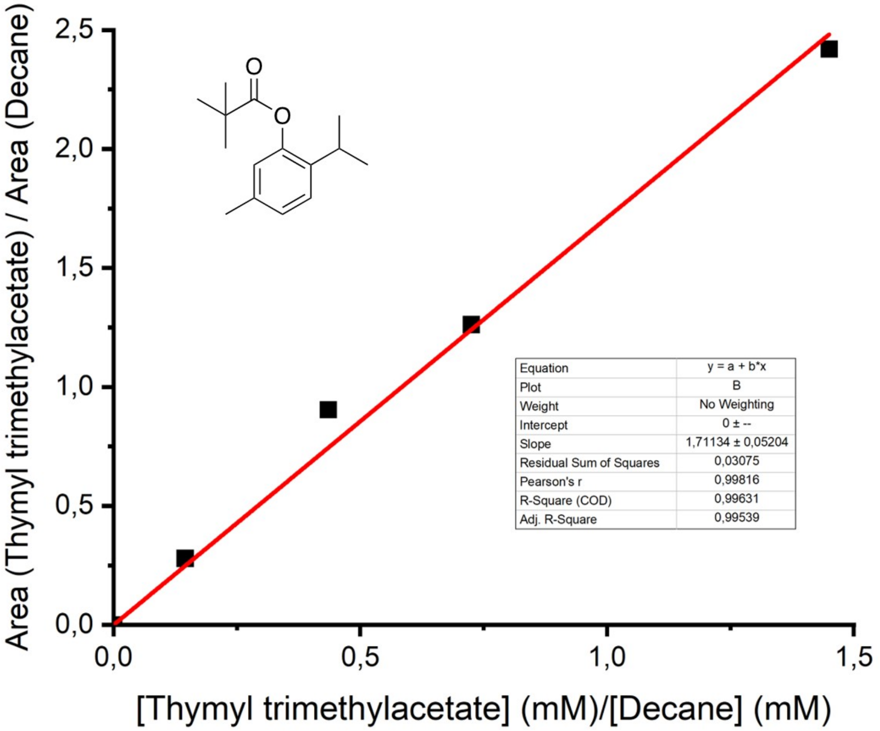

Supplementary Figure 30. 3c calibration curve using 10 mM decane as internal standard.

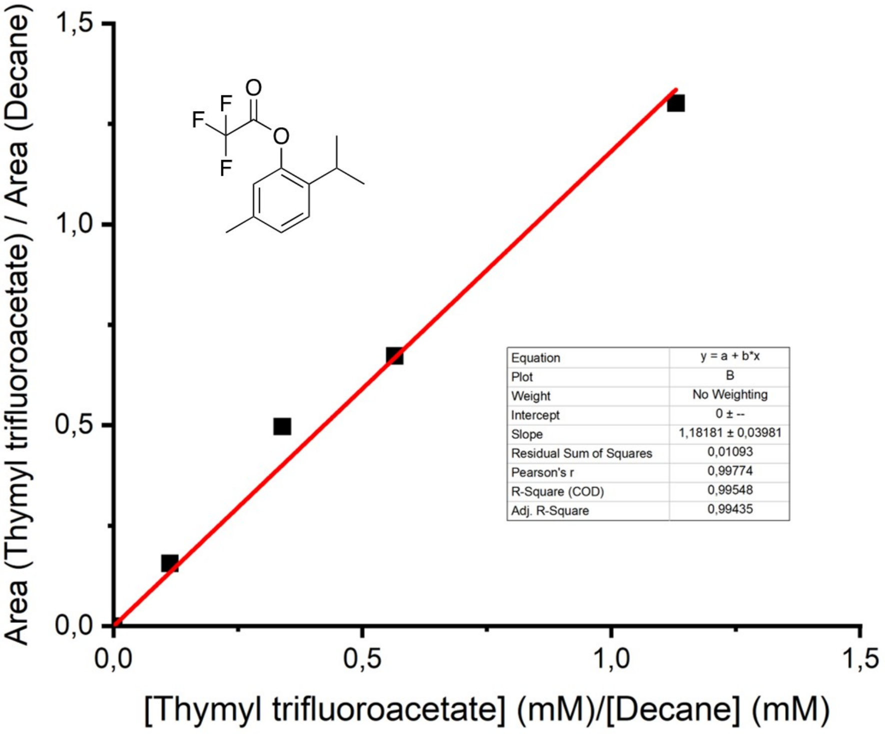

Supplementary Figure 31. 3d calibration curve using 10 mM decane as internal standard.

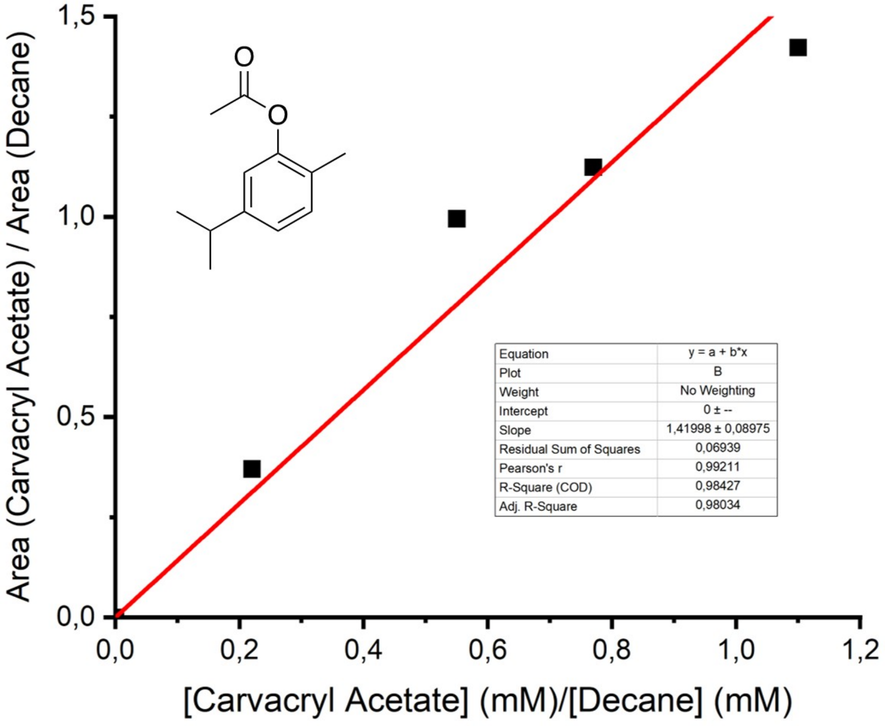

Supplementary Figure 32. 5a calibration curve using 10 mM decane as internal standard.

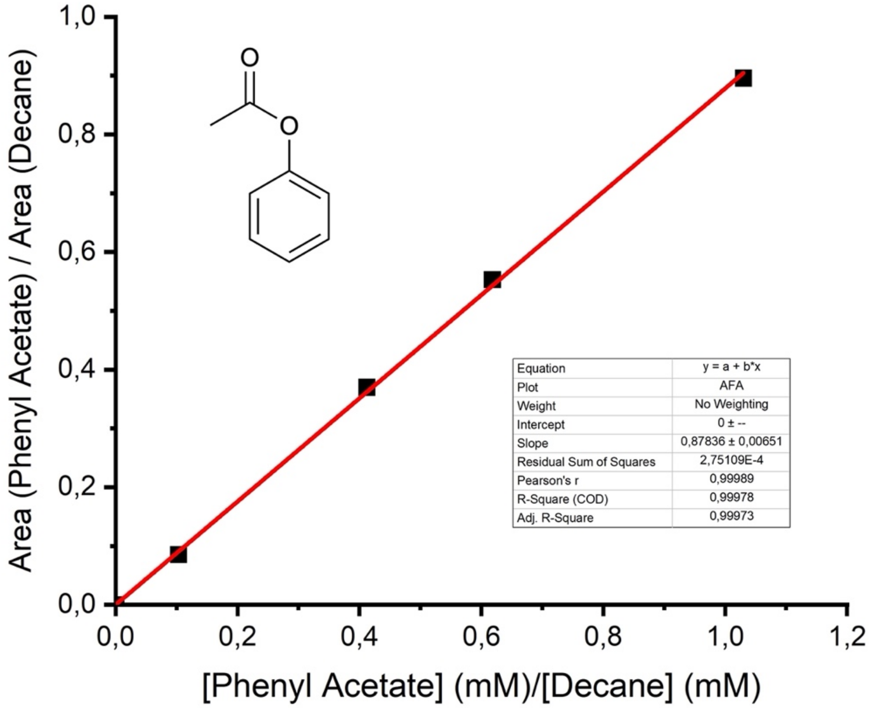

Supplementary Figure 33. 5b calibration curve using 10 mM decane as internal standard.

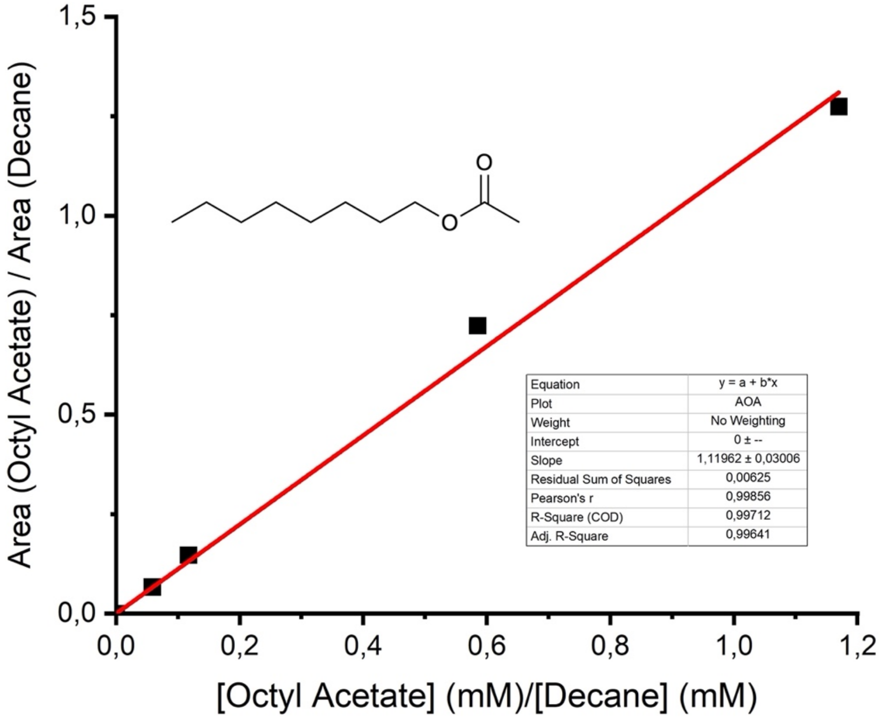

Supplementary Figure 34. 5c calibration curve using 10 mM decane as internal standard.

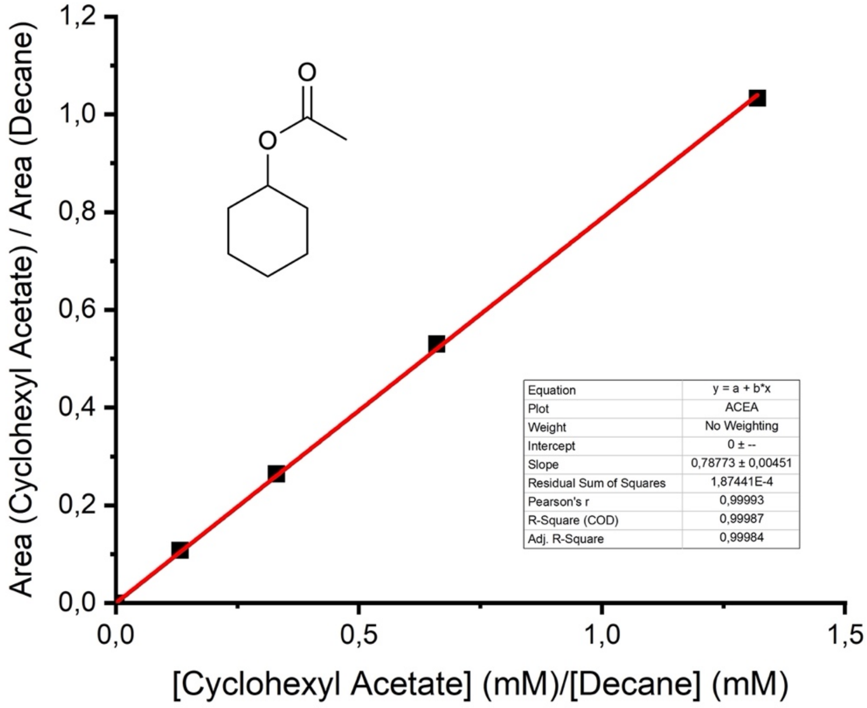

Supplementary Figure 35. 5d calibration curve using 10 mM decane as internal standard.

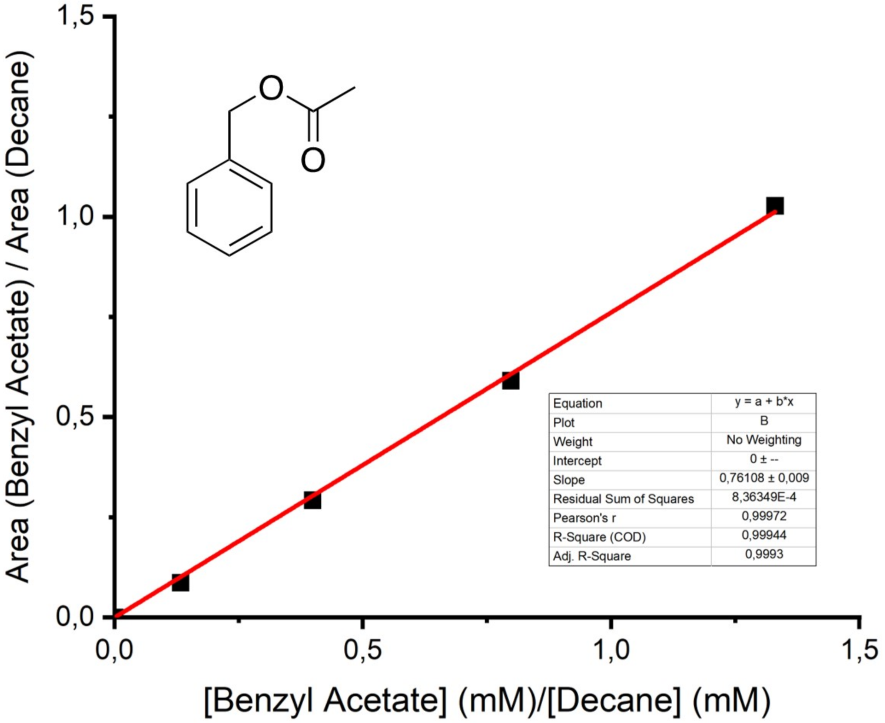

Supplementary Figure 36. 5e calibration curve using 10 mM decane as internal standard.

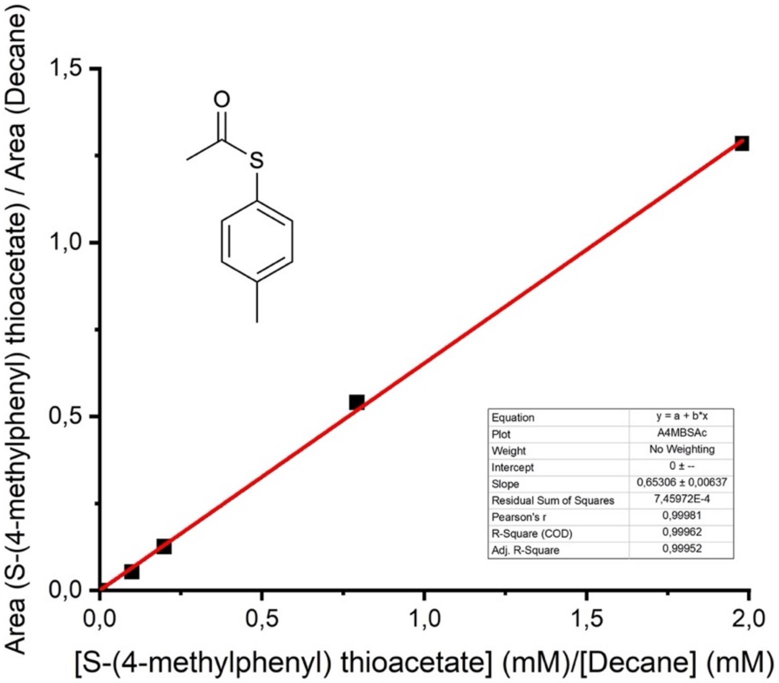

Supplementary Figure 37. 5f calibration curve using 10 mM decane as internal standard.

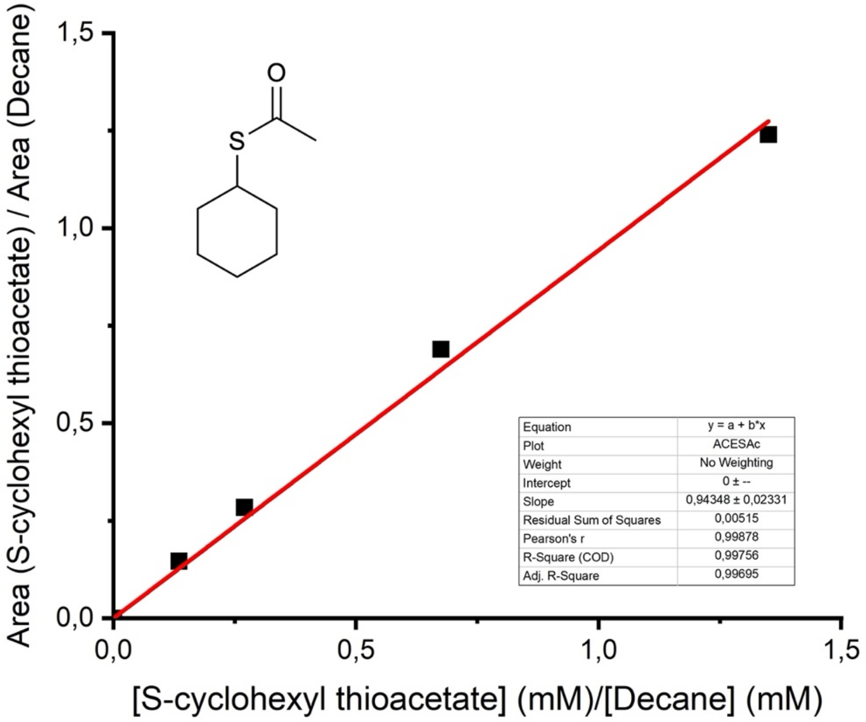

Supplementary Figure 38. 5g calibration curve using 10 mM decane as internal standard.

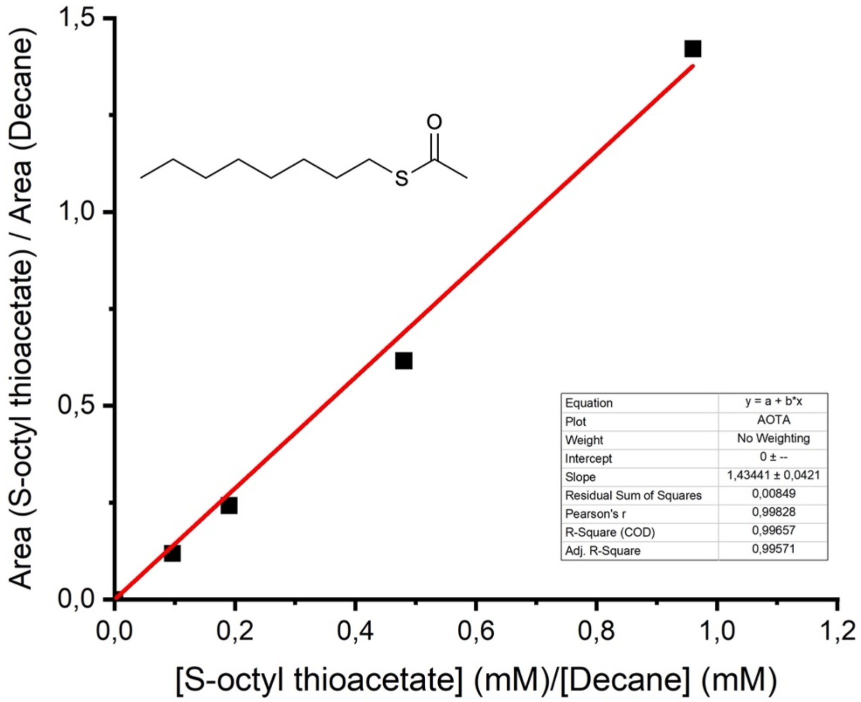

Supplementary Figure 39. 5h calibration curve using 10 mM decane as internal standard.

6. References

1. Tharamak, S., Yooboon, T., Pengsook, A., Ratwatthananon, A., Kumrungsee, N., Bullangpoti, V. and Pluempanupata, W. (2020). Synthesis of thymyl esters and their insecticidal activity against *Spodoptera litura* (Lepidoptera: Noctuidae). *Pest. Manag. Sci*. 76, 928-935. doi: 10.1002/ps.5598
2. Barros Silva, V., Lima Travassos, D., Nepel, A., Barison, A., Vilaça Costa, E., Scotti, et al. (2017). Synthesis and Chemometrics of Thymol and Carvacrol Derivatives as Larvicides against *Aedes aegypti*. *J. Arthropod. Borne Dis*. 11, 315–330.
3. Jain, I., Sharma, R. and Malik, P. (2019). Manganese-mediated acetylation of alcohols, phenols, thiols, and amines utilizing acetic anhydride. *Synth. Commun*. 49, 2952–2960. doi: 10.1080/00397911.2019.1650282.
4. Kuciński, K. and Hreczycho, G. (2018). S-Acetylation of Thiols Mediated by Triflic Acid: A Novel Route to Thioesters. *Org. Process Res. Dev*. 22, 489−493. doi: 10.1021/acs.oprd.7b00378.
